# Cutting-Edge Hydrogel Technologies in Tissue Engineering and Biosensing: An Updated Review

**DOI:** 10.3390/ma17194792

**Published:** 2024-09-29

**Authors:** Nargish Parvin, Vineet Kumar, Sang Woo Joo, Tapas Kumar Mandal

**Affiliations:** School of Mechanical Engineering, Yeungnam University, Gyeongsan 38541, Republic of Korea; nargish.parvin@gmail.com (N.P.); vineetfri@gmail.com (V.K.)

**Keywords:** hydrogel technologies, tissue engineering applications, biosensing innovations, 3D printing hydrogels, wearable health devices

## Abstract

Hydrogels, known for their unique ability to retain large amounts of water, have emerged as pivotal materials in both tissue engineering and biosensing applications. This review provides an updated and comprehensive examination of cutting-edge hydrogel technologies and their multifaceted roles in these fields. Initially, the chemical composition and intrinsic properties of both natural and synthetic hydrogels are discussed, highlighting their biocompatibility and biodegradability. The manuscript then probes into innovative scaffold designs and fabrication techniques such as 3D printing, electrospinning, and self-assembly methods, emphasizing their applications in regenerating bone, cartilage, skin, and neural tissues. In the realm of biosensing, hydrogels’ responsive nature is explored through their integration into optical, electrochemical, and piezoelectric sensors. These sensors are instrumental in medical diagnostics for glucose monitoring, pathogen detection, and biomarker identification, as well as in environmental and industrial applications like pollution and food quality monitoring. Furthermore, the review explores cross-disciplinary innovations, including the use of hydrogels in wearable devices, and hybrid systems, and their potential in personalized medicine. By addressing current challenges and future directions, this review aims to underscore the transformative impact of hydrogel technologies in advancing healthcare and industrial practices, thereby providing a vital resource for researchers and practitioners in the field.

## 1. Introduction

### 1.1. Overview of Hydrogels

Hydrogels are three-dimensional, hydrophilic polymer networks capable of retaining substantial amounts of water while maintaining their structure. This unique property is attributed to their cross-linked polymer chains, which can be either synthetic or natural. Synthetic hydrogels, such as polyvinyl alcohol (PVA) and polyethylene glycol (PEG), offer controlled and reproducible properties, whereas natural hydrogels like alginate, chitosan, and hyaluronic acid are biocompatible and biodegradable, making them suitable for biomedical applications [1,2]. Their versatility in water absorption and mechanical properties enables them to mimic natural tissue environments, which is critical for their application in tissue engineering and biosensing.

The ability of hydrogels to undergo significant swelling and deswelling in response to environmental stimuli (such as pH, temperature, and ionic strength) makes them ideal candidates for various biomedical applications. Advances in hydrogel technology have led to the development of “smart” hydrogels that respond to specific physiological conditions, enhancing their functionality in targeted drug delivery, wound healing, and as scaffolds for tissue engineering [3,4].

### 1.2. Importance in Tissue Engineering

Tissue engineering aims to develop functional constructs that restore, maintain, or improve damaged tissues or organs. Hydrogels play a pivotal role in this field due to their high water content, biocompatibility, and tunable mechanical properties that can mimic the extracellular matrix (ECM) of natural tissues [5]. These properties enable hydrogels to provide a supportive environment for cell proliferation and differentiation, which is essential for tissue regeneration.

One of the significant advancements in tissue engineering involves the use of 3D printing technology to fabricate hydrogel-based scaffolds with precise architecture. This technique allows for the creation of complex structures that can support cell growth and tissue formation [6]. Additionally, hydrogels can be engineered to release growth factors and other bioactive molecules in a controlled manner, further promoting tissue regeneration [7].

In bone tissue engineering, for example, hydrogels are used to create scaffolds that facilitate the growth of osteoblasts and the formation of new bone tissue [8]. Similarly, in cartilage tissue engineering, hydrogels provide a suitable environment for chondrocytes, promoting the regeneration of cartilage tissue [9]. The use of hydrogels in neural tissue engineering is also gaining attention, where they support the growth and differentiation of neural stem cells, aiding in the repair of nerve injuries [10].

### 1.3. Relevance in Bio-Sensing

Hydrogels have emerged as crucial components in the development of biosensors due to their ability to respond to various biological and chemical stimuli. Their high water content and porous structure allow for the diffusion of analytes, making them suitable for detecting a wide range of biomolecules [11,12]. Hydrogels can be functionalized with specific recognition elements, such as enzymes, antibodies, or nucleic acids, enabling them to selectively bind to target molecules and produce a detectable signal.

**Figure 1:**
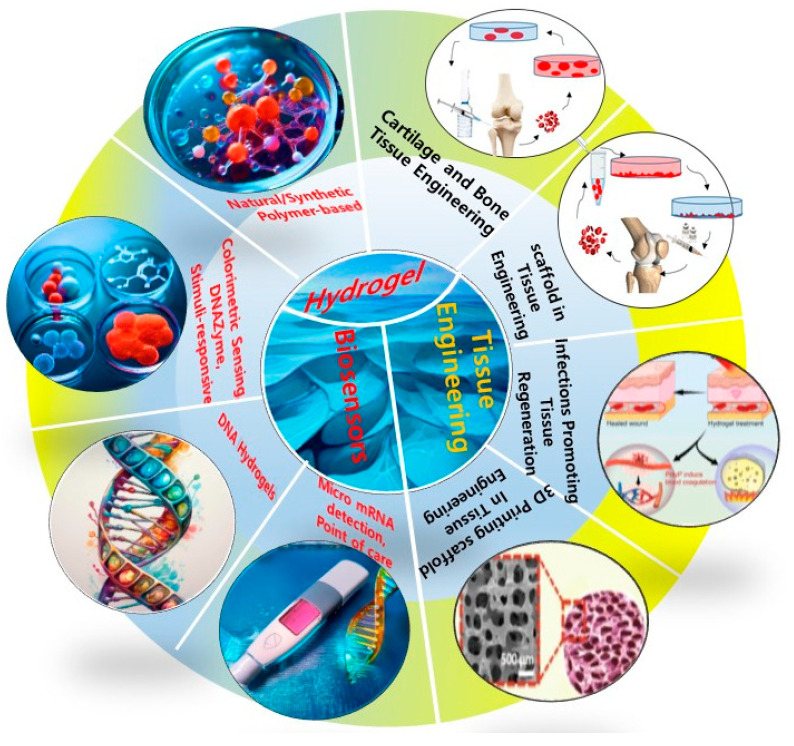
Illustration depicting different designs of hydrogel biosensors and tissue engineering applications, along with their respective functional roles.

In medical diagnostics, hydrogel-based biosensors are used for the continuous monitoring of glucose levels in diabetic patients. These sensors can detect changes in glucose concentration and provide real-time data, improving disease management [13]. Similarly, hydrogel-based sensors are used for the detection of pathogens and biomarkers, contributing to early diagnosis and treatment of infectious diseases and cancer [14]. Figure 1 illustrates the diverse designs of hydrogel biosensors and their applications in tissue engineering, highlighting their functional roles in these fields. Hydrogel biosensors leverage the unique properties of hydrogels, such as high water content and biocompatibility, to create responsive platforms for detecting biological signals. These sensors can be engineered to react to specific stimuli, such as pH changes or the presence of biomolecules, enabling real-time monitoring of physiological conditions. In tissue engineering, hydrogels serve as scaffolds that mimic the extracellular matrix, promoting cell adhesion, proliferation, and differentiation. The integration of bioactive molecules within these hydrogels can enhance their functionality, making them suitable for regenerative medicine applications. The figure emphasizes the versatility of hydrogels in combining sensing capabilities with biological support, paving the way for advanced therapeutic strategies and improved patient outcomes. This dual functionality is particularly beneficial for applications such as wound healing, where both biosensing and tissue regeneration are crucial for effective treatment. Environmental monitoring is another area where hydrogel-based sensors are making an impact. These sensors can detect pollutants and toxins in water and air, providing valuable information for environmental protection and public health [15]. The development of smart hydrogels that can change their properties in response to specific environmental conditions further enhances their application in biosensing [16,17].

### 1.4. Objectives of the Review

This review aims to provide a comprehensive and updated examination of the latest advancements in hydrogel technologies, focusing on their applications in tissue engineering and biosensing. By exploring the chemical composition, fabrication techniques, and functional properties of hydrogels, the review highlights their potential in various biomedical and environmental applications. Key challenges and future directions in the field will also be discussed to provide insights for researchers and practitioners.

The objectives of this review include the following:

Summarizing the fundamental properties of hydrogels and their relevance to tissue engineering and biosensing.

Discuss recent advancements in the design and fabrication of hydrogel-based scaffolds and sensors.

Exploring the applications of hydrogels in various areas of tissue engineering, including bone, cartilage, skin, and neural tissue regeneration.

Examining the role of hydrogels in the development of biosensors for medical diagnostics and environmental monitoring.

Identifying current challenges and future research directions to advance the field of hydrogel technologies.

By achieving these objectives, this review aims to provide a valuable resource for researchers, clinicians, and industry professionals involved in the development and application of hydrogel technologies in tissue engineering and biosensing.

## 2. Hydrogel Fundamentals

### 2.1. Chemical Composition and Properties

Hydrogels are composed of hydrophilic polymer chains that are cross-linked to form a three-dimensional network capable of retaining large amounts of water. This fundamental property of hydrogels is due to their chemical composition and structure, which can be categorized into natural and synthetic hydrogels.

#### 2.1.1. Natural Hydrogels

Natural hydrogels are derived from biological sources, making them inherently biocompatible and often biodegradable. Common natural hydrogels include the following:

**1. Alginate**: Alginate, a naturally occurring polysaccharide extracted from brown seaweed, forms hydrogels through ionic cross-linking with divalent cations, such as calcium ions. This mild gelation process makes alginate highly suitable for biomedical applications, especially in wound dressings and tissue engineering. Its biocompatibility, non-toxic nature, and ability to gel under physiological conditions without requiring harsh chemicals are significant advantages [18]. In wound care, alginate hydrogels help maintain a moist environment, absorb exudates, and promote healing by providing a scaffold for cellular infiltration and tissue regeneration. In tissue engineering, alginate serves as an excellent matrix for encapsulating cells, supporting cell proliferation and tissue formation. However, one limitation is its lack of cell-adhesive properties, which can be addressed by modifying the polymer or incorporating bioactive molecules to enhance cell interactions. Alginate’s versatility, ease of gelation, and tunable properties continue to drive its widespread use in various regenerative medicine and drug delivery applications.

**2. Chitosan**: Chitosan, derived from chitin found in the exoskeletons of crustaceans, is widely used in hydrogel formulations due to its unique antibacterial properties and biodegradability. Its biocompatibility and ability to degrade into non-toxic byproducts make it an attractive material for biomedical applications. Chitosan hydrogels are particularly valuable in wound healing, where their antibacterial action helps reduce infection risk while promoting tissue regeneration. The hydrogels also maintain a moist environment, which is essential for efficient wound closure. In drug delivery systems, chitosan hydrogels offer controlled and sustained release of therapeutic agents, enhancing the effectiveness of treatments [19]. However, the material’s poor solubility in neutral or basic pH environments can limit its use, which can be addressed through chemical modifications to improve its solubility and mechanical strength. Overall, chitosan hydrogels are promising in regenerative medicine due to their bioactivity, biodegradability, and versatility in various biomedical applications.

**3. Hyaluronic Acid (HA)**: Hyaluronic acid (HA) is a naturally occurring polysaccharide integral to the extracellular matrix, known for its remarkable biocompatibility and hydrophilicity. Its ability to retain moisture makes HA hydrogels particularly valuable in cosmetic applications, where they are employed for dermal fillers and skin rejuvenation therapies. In tissue engineering, HA serves as a scaffold that not only supports cellular adhesion and proliferation but also facilitates the migration of cells crucial for tissue regeneration. The viscoelastic properties of HA hydrogels can be tailored through chemical modification, allowing for the development of materials that mimic the mechanical behavior of native tissues. Additionally, HA’s intrinsic bioactivity promotes cellular signaling and tissue repair processes, making it an excellent candidate for applications in wound healing and cartilage regeneration. However, challenges such as rapid enzymatic degradation in vivo can limit the longevity of HA hydrogels, necessitating the incorporation of cross-linking agents or alternative delivery systems to enhance their stability. Overall, HA hydrogels represent a versatile platform in regenerative medicine and cosmetic applications, combining biocompatibility with functional properties tailored to specific therapeutic needs [20].

**4. Collagen**: Collagen, the most abundant protein in mammals, serves as a fundamental component of the extracellular matrix (ECM), providing structural integrity and biochemical signals essential for cell behavior. Collagen-based hydrogels capitalize on its inherent biocompatibility, allowing for excellent cellular adhesion and proliferation. These hydrogels are particularly advantageous for applications in skin and cartilage repair, as they closely mimic the native ECM, promoting the natural healing processes [21]. The tunable mechanical properties of collagen hydrogels can be achieved through varying concentrations, cross-linking methods, and incorporation of other biomaterials, enabling customization for specific tissue engineering needs. Moreover, collagen’s bioactive peptides can enhance cellular responses, facilitating migration, differentiation, and matrix deposition. However, the rapid degradation of collagen in vivo poses challenges, particularly in load-bearing applications, where maintaining structural integrity over time is crucial. Strategies to stabilize collagen hydrogels include chemical modifications, hybridization with synthetic polymers, or the incorporation of bioactive molecules that slow down degradation rates. Overall, collagen-based hydrogels represent a promising approach in regenerative medicine, providing a biocompatible and biomimetic environment conducive to tissue repair and regeneration.

**5. Gelatin**: Gelatin, a denatured form of collagen, retains many of the advantageous properties of its precursor, making it a widely used biomaterial in tissue engineering and drug delivery applications. Its natural origin ensures excellent biocompatibility, promoting cell adhesion, proliferation, and differentiation, which are critical for effective tissue regeneration. Gelatin hydrogels can be synthesized through various methods, including physical and chemical cross-linking, enabling the tailoring of their mechanical properties and degradation rates to meet specific application needs. The biodegradability of gelatin is a significant advantage, as it breaks down into non-toxic byproducts, allowing for gradual replacement by newly formed tissue. Moreover, gelatin’s inherent bioactivity, attributed to its bioactive peptides, can facilitate cellular responses essential for tissue repair. However, gelatin hydrogels may face challenges related to mechanical strength and stability, especially in load-bearing applications. To address these limitations, researchers often combine gelatin with other polymers or incorporate reinforcement materials, such as nanoparticles or fibers, to enhance their structural integrity. Overall, gelatin hydrogels represent a versatile and effective option in regenerative medicine, providing a supportive matrix that can be easily modified to accommodate various therapeutic strategies, including drug delivery and tissue engineering [22].

#### 2.1.2. Synthetic Hydrogels

Synthetic hydrogels are created from synthetic polymers, allowing for more controlled and tunable properties. Some commonly used synthetic hydrogels include the following:

**1. Polyethylene Glycol (PEG)**: Polyethylene glycol (PEG) is a synthetic polymer widely recognized for its biocompatibility, hydrophilicity, and resistance to protein adsorption, making it an attractive material for a variety of biomedical applications, particularly in drug delivery and tissue engineering [23]. PEG hydrogels are formed through cross-linking processes that create a three-dimensional network capable of holding large amounts of water, which mimics the natural extracellular matrix (ECM). This water retention not only supports cell proliferation but also facilitates the diffusion of nutrients and waste, critical for tissue regeneration. PEG’s resistance to protein adsorption reduces the likelihood of nonspecific binding, which is particularly valuable in drug delivery systems, as it helps to avoid premature immune recognition and prolongs the circulation time of drug-loaded carriers. Moreover, PEG hydrogels offer tunable mechanical and chemical properties by adjusting the molecular weight and cross-linking density, allowing them to be tailored for specific applications, such as creating softer matrices for cartilage regeneration or stiffer scaffolds for bone repair. However, despite its numerous advantages, PEG lacks inherent biological activity, necessitating functionalization with bioactive molecules like peptides or growth factors to enhance cell attachment and integration. This tunability, combined with its inert nature and safety profile, makes PEG a versatile material in advanced biomedical applications, although challenges such as its slow degradation rate and limited bioactivity continue to be areas of active research.

**2. Polyvinyl Alcohol (PVA)**: Polyvinyl alcohol (PVA) hydrogels are synthetic materials renowned for their high mechanical strength, flexibility, and excellent biocompatibility, which make them well suited for various biomedical applications, including contact lenses, wound dressings, and tissue engineering scaffolds [24]. Hydrogels are typically produced through a freeze–thaw cycling method, which induces physical cross-linking without the need for chemical agents, thus minimizing potential toxicity. The resulting hydrogel has a highly elastic structure that can retain significant amounts of water, closely mimicking the hydration properties of soft tissues. In applications such as contact lenses, PVA hydrogels offer clear advantages due to their transparency, oxygen permeability, and ability to maintain moisture, which improves user comfort and prevents dry eye syndrome. In wound dressings, PVA hydrogels provide a moist environment that promotes healing, while their mechanical properties protect the wound site from external contaminants. Furthermore, PVA’s biocompatibility and low immunogenicity reduce the risk of adverse reactions when applied to sensitive or damaged tissues. Despite these benefits, PVA hydrogels lack inherent bioactivity, similar to other synthetic polymers, necessitating surface modification or the incorporation of bioactive agents, such as antimicrobial peptides or growth factors, to enhance their functionality in tissue engineering applications. Additionally, while PVA hydrogels exhibit impressive mechanical properties, their non-biodegradability can limit their use in applications that require scaffold degradation in concert with tissue regeneration, thus motivating ongoing research to modify PVA for improved biodegradability and enhanced biological interaction.

**3. Polyacrylamide (PAAm)**: Polyacrylamide (PAAm) hydrogels are highly valued for their outstanding mechanical properties, high water absorption capacity, and versatility, making them widely used in applications ranging from agriculture to superabsorbent materials [25]. Am hydrogels are formed through the polymerization of acrylamide monomers, resulting in a cross-linked network that can absorb and retain large quantities of water relative to its dry weight, a property that makes them particularly effective as soil conditioners in agriculture. When incorporated into soil, PAAm hydrogels enhance water retention, reducing the need for frequent irrigation and improving drought resistance for crops. This ability to hold water and release it gradually aligns with sustainable farming practices, especially in arid regions where water conservation is crucial. Additionally, PAAm hydrogels are employed in industrial applications as superabsorbent materials in products like diapers, hygiene pads, and wound dressings, where their capacity to manage moisture plays a critical role in performance. The hydrophilic nature of PAAm allows it to maintain a moist environment, which is beneficial for wound healing as it prevents tissue desiccation. However, the major challenge with PAAm hydrogels is their non-biodegradability, which raises environmental concerns when used in large-scale agricultural or disposable consumer products. Furthermore, PAAm itself can be cytotoxic in its monomeric form, and therefore careful polymerization and purification are required to ensure the safety of the hydrogel for biological applications. Ongoing research aims to improve the biodegradability and reduce the environmental footprint of PAAm hydrogels while retaining their beneficial absorbent properties and mechanical strength for broad industrial and biomedical applications.

**4. Poly(2-hydroxyethyl methacrylate) (pHEMA)**: Poly(2-hydroxyethyl methacrylate) (pHEMA) hydrogels are widely utilized in biomedical applications, particularly in the development of contact lenses and drug delivery systems, due to their excellent biocompatibility, optical clarity, and hydrophilic nature [26]. pHEMA is synthesized through free radical polymerization, resulting in a hydrogel that is highly transparent, making it ideal for ophthalmic uses such as soft contact lenses. The material’s ability to absorb water while maintaining structural integrity ensures that contact lenses made from pHEMA remain hydrated, providing comfort to users by reducing dryness and irritation. Moreover, the optical clarity of pHEMA allows for unobstructed vision, making it a preferred choice in vision correction. In drug delivery systems, pHEMA hydrogels serve as carriers capable of encapsulating and gradually releasing therapeutic agents over time. Their tunable porosity allows for controlled drug release rates, making pHEMA suitable for sustained drug delivery applications where a constant therapeutic dose is required. Additionally, pHEMA hydrogels exhibit low protein adsorption, reducing the risk of fouling or immune response, which is particularly important in long-term biomedical implants. However, pHEMA hydrogels have limitations, particularly in terms of their relatively low mechanical strength and lack of inherent bioactivity. These properties often necessitate the incorporation of other materials or bioactive molecules to enhance cell interaction and mechanical performance, especially in tissue engineering applications. Despite these challenges, pHEMA continues to be a cornerstone in the field of biomaterials due to its proven safety, effectiveness, and versatility in medical applications.

Table 1 presents a comprehensive overview of various hydrogel synthesis processes, highlighting the comparative results alongside their respective advantages and disadvantages. This table systematically categorizes different hydrogel types based on their synthesis methods, such as ionotropic gelation, enzymatic cross-linking, and photopolymerization. Illustrating the unique characteristics and performance outcomes associated with each method allows for an informed understanding of how these factors influence the applicability of hydrogels in diverse fields, including tissue engineering and drug delivery. The inclusion of both benefits and limitations further aids in the evaluation of material selection for specific applications, providing a nuanced perspective essential for advancing hydrogel technology. Hydrogels represent a class of materials that exhibit significant potential in biomedical applications due to their unique properties, including high water retention and biocompatibility. The synthesis processes of hydrogels vary significantly, leading to distinct mechanical and biological characteristics that influence their application. For example, natural hydrogels like alginate are synthesized via ionotropic gelation, which yields biocompatible structures suitable for drug delivery and tissue engineering. While alginate’s high water content is beneficial, its limited mechanical strength can be a drawback in load-bearing applications [27]. Similarly, collagen hydrogels, formed through enzymatic cross-linking, not only mimic the extracellular matrix but also support cell proliferation; however, their variability in sourcing and cost can pose challenges [28]. On the synthetic side, polyethylene glycol (PEG) hydrogels created through photopolymerization are notable for their tunable properties and low toxicity, making them ideal for applications in 3D bioprinting [29]. Moreover, poly(N-isopropylacrylamide) (PNIPAAm) hydrogels showcase temperature-responsive behavior, making them particularly advantageous for smart drug delivery systems [30]. This variety in synthesis methods not only defines the performance characteristics of hydrogels but also necessitates careful consideration of their specific applications to optimize efficacy and safety [31]. The ongoing advancements in hydrogel technology continue to expand their functional versatility, paving the way for innovative solutions in tissue engineering, drug delivery, and regenerative medicine.

**5. Poly(N-isopropylacrylamide) (PNIPAM)**: Poly(N-isopropylacrylamide) (PNIPAM) hydrogels are thermoresponsive materials known for their sharp phase transition at a specific temperature, typically around 32 °C, making them highly valuable in applications such as drug delivery and tissue engineering [32]. PNIPAM hydrogels undergo a reversible change from a swollen, hydrated state to a collapsed, dehydrated state when exposed to temperatures above their lower critical solution temperature (LCST). This unique property allows PNIPAM to release encapsulated drugs or bioactive molecules in a controlled manner, triggered by slight temperature variations, which is particularly advantageous in targeted drug delivery systems where temperature changes can be utilized for localized treatment, such as in hyperthermia cancer therapies. The phase transition also makes PNIPAM hydrogels suitable for tissue engineering, as they can be designed to release growth factors or cells in response to the body’s physiological temperature. Their ability to respond to environmental stimuli without requiring external chemical triggers further enhances their versatility for in vivo applications. However, while PNIPAM is widely researched for its smart material properties, it has certain limitations, including poor biocompatibility in its unmodified form and a lack of biodegradability, which may pose challenges for long-term biomedical use. To address these concerns, PNIPAM is often modified or combined with other biocompatible polymers to improve its biological performance and ensure safety in clinical applications. Additionally, the sharpness of its phase transition can be fine-tuned by altering the polymer’s structure, offering the possibility of custom-tailored hydrogels for specific medical needs. Overall, PNIPAM’s thermoresponsive behavior and adaptability make it a promising material for cutting-edge biomedical technologies.

### 2.2. Physical and Mechanical Properties

The physical and mechanical properties of hydrogels are crucial for their application in various fields, including tissue engineering and biosensing. These properties can be tailored by altering the polymer composition, cross-linking density, and environmental conditions.

**1. Swelling Behavior**: Swelling behavior is a fundamental property of hydrogels that directly impacts their functionality in various biomedical and industrial applications. The ability of hydrogels to absorb and retain water is largely determined by the hydrophilicity of the polymer chains, the cross-linking density within the network, and the ionic strength of the surrounding environment [33]. Hydrophilic polymers, which contain functional groups such as hydroxyl, carboxyl, or amide groups, readily interact with water molecules, allowing the hydrogel to take up significant amounts of water, leading to swelling. However, the extent of swelling is inversely related to the cross-linking density of the hydrogel. Hydrogels with a higher cross-linking density have more tightly bound polymer chains, which restricts the amount of water that can be absorbed, thereby reducing swelling. While this decreased swelling often enhances the mechanical strength and structural stability of the hydrogel, it may also limit its flexibility and capacity to encapsulate or release therapeutic agents in biomedical applications. Moreover, the ionic strength of the surrounding medium plays a crucial role, as higher ionic concentrations can lead to reduced swelling due to osmotic pressure balancing between the hydrogel and its environment. This is particularly relevant in physiological conditions, where the presence of salts and other ions influences the swelling behavior of hydrogels used in tissue engineering, drug delivery, and wound healing. Optimizing the balance between swelling capacity and mechanical strength is key for designing hydrogels tailored for specific applications, ensuring they provide sufficient hydration while maintaining structural integrity under different environmental conditions.

**Figure 2:**
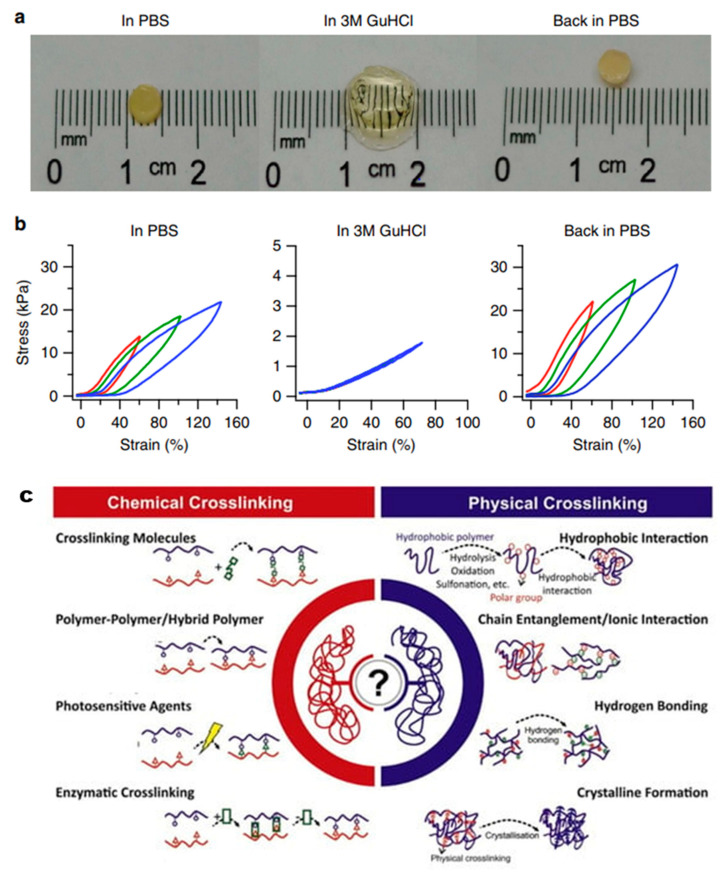
(**a**,**b**) Overview of the physical and mechanical characteristics of (FL) hydrogels. Reprinted with permission from [35]. https://doi.org/10.1038/ncomms3974. (**c**) Diagram illustrating the different types of physical and chemical cross-linking methods used in hydrogels (adapted from [36], CC-BY license) https://doi.org/10.3390/gels7040255.

**2. Mechanical Strength**: The mechanical properties of hydrogels, such as elasticity, tensile strength, and compressive modulus, are vital for their use in load-bearing applications. Synthetic hydrogels like PVA and PEG can be engineered to achieve desired mechanical properties by adjusting the cross-linking density and polymer concentration [34]. Figure 2a,b provides a comprehensive overview of the physical and mechanical properties of (FL) hydrogels. It highlights key attributes such as the gel’s elasticity, compressive strength, and swelling behavior, which are critical for evaluating its suitability in various applications. The figure details how these properties vary with changes in formulation or external conditions, offering insights into the hydrogel’s performance under different stress conditions. This information is crucial for optimizing (FL) hydrogels for specific uses, such as in biomedical applications where mechanical stability and flexibility are essential [35]. Figure 2c provides a comprehensive overview of the various physical and chemical cross-linking methods utilized in hydrogel synthesis. This diagram highlights the distinction between these two categories, with physical cross-linking typically involving non-covalent interactions such as hydrogen bonding, ionic interactions, and hydrophobic effects, which contribute to the stability and responsiveness of the hydrogel. In contrast, chemical cross-linking relies on covalent bonds, resulting in more robust and durable structures. The versatility of these cross-linking strategies allows for fine-tuning of hydrogel properties, including mechanical strength, swelling behavior, and degradation rates, which are critical for applications in tissue engineering, drug delivery, and biosensing. The illustration effectively conveys the complexity and adaptability of hydrogel design, showcasing how different cross-linking methods can be strategically employed to meet specific functional requirements in biomedical applications [36].

**3. Degradation Rate**: The degradation rate of hydrogels is a crucial factor in their design, especially for biomedical applications like tissue engineering, where the material must degrade in sync with the formation of new tissue [37]. Hydrogels are engineered with biodegradable linkages within their polymer network, which break down over predetermined time frames in response to biological or environmental conditions, such as enzymatic activity, hydrolysis, or pH changes. These biodegradable hydrogels allow for a gradual dissolution of the scaffold, ensuring that as new tissue forms and matures, the scaffold disappears without causing harm or requiring surgical removal. This controlled degradation is essential in applications like wound healing and regenerative medicine, where the material should provide temporary support before being replaced by natural tissue. The rate of degradation can be tailored by adjusting the composition of the polymer backbone, the type of cross-linkers used, or by introducing specific functional groups that respond to biological cues. For instance, faster-degrading hydrogels may be ideal for short-term applications such as drug delivery, where the rapid release of therapeutics is needed, while slower-degrading scaffolds are more suitable for long-term tissue regeneration. A major challenge is achieving a balance between degradation rate and mechanical integrity; the scaffold must maintain its structural function long enough to support tissue growth without degrading prematurely. Advances in material science have allowed for precise tuning of degradation kinetics, enhancing the functionality of hydrogels in personalized medicine and tissue repair applications.

**4. Porosity**: Porosity is a critical feature in the design of hydrogels, especially for tissue engineering, as it directly influences the material’s ability to support cell infiltration, nutrient diffusion, and waste removal [38]. The size, distribution, and interconnectivity of pores within the hydrogel matrix are essential for promoting cell viability and function, as they create pathways for the exchange of nutrients, oxygen, and metabolic waste. Large, well-distributed pores enhance the diffusion of essential molecules and provide spaces for cells to proliferate and migrate, which is crucial in the development of tissue scaffolds. Techniques such as freeze-drying and gas foaming are commonly employed to generate hydrogels with controlled porosity. Freeze-drying, for example, allows for the creation of highly porous structures by freezing the hydrogel solution and then sublimating the solvent, leaving behind a network of pores. Gas foaming, on the other hand, introduces gaseous agents that expand within the polymer matrix, forming pores of specific sizes. These techniques enable fine-tuning of pore dimensions, allowing researchers to optimize hydrogels for different tissue types or therapeutic applications. Hydrogels with smaller pore sizes are often used for drug delivery systems, where controlled release is required, while larger pores are more suitable for tissue engineering applications, such as bone and cartilage regeneration, where cell penetration and vascularization are critical. However, achieving the right balance between porosity and mechanical strength remains a challenge, as highly porous hydrogels may be mechanically weak, compromising their structural integrity. Thus, advancements in hydrogel fabrication techniques aim to create scaffolds that offer both adequate porosity and sufficient mechanical support to mimic the natural extracellular matrix effectively.

**5. Stimuli-Responsive Properties**: Smart hydrogels are an advanced class of materials that can respond to external stimuli like temperature, pH, light, and even electric fields, making them highly versatile for biomedical applications [39]. Among them, poly(N-isopropylacrylamide) (PNIPAM) hydrogels are well known for their thermoresponsive behavior, undergoing a sharp and reversible phase transition around 32 °C, which is close to body temperature. Below this critical temperature, PNIPAM hydrogels are in a swollen, hydrated state, while above this threshold, they collapse into a dehydrated form. This phase transition makes PNIPAM particularly suitable for controlled drug delivery, as it allows for the precise release of therapeutics triggered by slight changes in temperature, such as those associated with body heat or localized hyperthermia in cancer treatments. Additionally, PNIPAM’s ability to switch between states can be leveraged for cell encapsulation, where the material can encapsulate cells in their swollen state and release them in response to temperature changes, enabling targeted therapies or tissue engineering applications. Beyond temperature, smart hydrogels can also be engineered to respond to pH variations, which is valuable in areas such as wound healing or drug delivery in acidic environments like tumors. Light-responsive hydrogels further extend the application scope, where light can be used to trigger gelation or degradation, offering non-invasive control over material behavior. However, challenges remain in ensuring the biocompatibility, degradation control, and mechanical stability of these smart hydrogels, particularly for in vivo applications, where complex environments may affect their responsiveness. Ongoing research focuses on improving the design of these materials to enhance their precision, responsiveness, and safety in clinical and industrial settings.

### 2.3. Biocompatibility and Biodegradability

Biocompatibility and biodegradability are essential characteristics of hydrogels, particularly for biomedical applications. These properties ensure that hydrogels do not elicit adverse immune responses and can be safely broken down and absorbed or excreted by the body.

**1. Biocompatibility**: Biocompatibility is a fundamental requirement for hydrogels used in medical applications, as these materials must interact with biological tissues without inducing adverse reactions such as toxicity, inflammation, or immune rejection [40]. Natural hydrogels like collagen, hyaluronic acid, and alginate are inherently biocompatible due to their resemblance to the extracellular matrix (ECM) and their ability to support cell adhesion, migration, and proliferation. These materials are commonly used in tissue engineering, wound healing, and drug delivery, where their biological properties align well with the body’s natural healing processes. However, the mechanical and degradation properties of natural hydrogels often need to be optimized for specific applications, leading to the development of synthetic hydrogels. While synthetic hydrogels, such as polyethylene glycol (PEG) and polyvinyl alcohol (PVA), offer greater control over mechanical strength, degradation rates, and structural properties, they do not naturally exhibit the same level of biocompatibility. Therefore, synthetic hydrogels are frequently modified through surface functionalization, copolymerization, or the incorporation of bioactive molecules to enhance their biocompatibility. For instance, synthetic hydrogels can be engineered to present cell-adhesive ligands, promote tissue integration, or degrade in response to specific biological cues, thus minimizing the risk of inflammatory or immune responses. Achieving a balance between biocompatibility and functional properties such as mechanical integrity, degradation, and responsiveness remains a key challenge. As the field advances, novel strategies like combining natural and synthetic components or employing bioactive coatings are being explored to improve the safety and efficacy of hydrogel-based biomedical devices, particularly for long-term implants and regenerative medicine.

**2. Biodegradability**: The ability of a hydrogel to degrade into non-toxic byproducts that can be metabolized or excreted by the body is crucial for temporary medical implants and drug delivery systems. Natural hydrogels are generally biodegradable, whereas synthetic hydrogels can be engineered to include biodegradable linkages [41]. Biodegradability is a critical property of hydrogels in the context of temporary medical implants and drug delivery systems, as it determines the material’s ability to break down into non-toxic byproducts that can be safely metabolized or excreted by the body. Natural hydrogels, such as collagen, gelatin, and alginate, are typically biodegradable due to their composition, which closely resembles the extracellular matrix (ECM) and can be enzymatically degraded by physiological processes. This intrinsic biodegradability allows them to provide temporary support for tissue regeneration while being gradually replaced by natural tissue. In contrast, synthetic hydrogels often lack inherent biodegradability; therefore, they are designed with specific biodegradable linkages within their polymer chains. By incorporating these linkages—such as ester or amide bonds—researchers can tailor the degradation rate of synthetic hydrogels to match the healing process of the tissue they support. For instance, hydrogels designed to degrade more rapidly may be appropriate for applications where swift tissue integration is necessary, while those that degrade slowly can provide long-term support in applications such as bone or cartilage engineering. The degradation products must be carefully selected to ensure they are non-toxic and do not provoke an inflammatory response. Advances in material chemistry have enabled the design of hydrogels with programmable degradation profiles that respond to specific biological stimuli, such as pH changes or enzymatic activity, allowing for more precise control over drug release rates and scaffold degradation. This synergy between biodegradability and functionality is essential for the development of effective hydrogel-based therapies that promote healing while minimizing complications associated with non-degradable materials. As research progresses, the challenge remains to achieve a balance between mechanical properties, degradation rates, and biocompatibility to optimize these materials for various clinical applications.

**3. Cellular Interactions**: For tissue engineering, hydrogels must support cellular adhesion, proliferation, and differentiation. The incorporation of bioactive motifs, such as RGD peptides, can enhance cell–hydrogel interactions and promote tissue integration [42]. Cellular interactions are paramount for the success of hydrogels in tissue engineering applications, as these materials must not only provide a physical scaffold but also actively support cellular adhesion, proliferation, and differentiation. The interaction between cells and the hydrogel matrix is mediated by various factors, including the chemical composition of the hydrogel and the presentation of bioactive motifs. For instance, the incorporation of specific peptides, such as arginine–glycine–aspartic acid (RGD) sequences, can significantly enhance cell–hydrogel interactions. RGD peptides are known to facilitate cell attachment by binding to integrin receptors on the cell surface, thereby promoting signaling pathways that drive cellular behaviors essential for tissue regeneration. Moreover, the spatial arrangement and density of these bioactive motifs can be fine-tuned to optimize cell responses, influencing processes such as migration and differentiation. Additionally, the mechanical properties of the hydrogel, including stiffness and elasticity, play a crucial role in mimicking the native extracellular matrix and guiding stem cell fate decisions. Hydrogels with suitable mechanical characteristics can promote stem cell differentiation into specific lineages, such as osteogenic or chondrogenic pathways, depending on the target tissue type. However, achieving a balance between adequate mechanical strength and bioactivity remains a challenge, as overly rigid scaffolds can hinder cell migration, while too soft scaffolds may not provide sufficient support. Ongoing research in the field aims to develop hybrid hydrogels that combine synthetic and natural components, leveraging the strengths of both to enhance cellular interactions while maintaining desirable mechanical properties. By optimizing the bioactivity of hydrogels through strategic incorporation of functional groups and peptides, researchers are paving the way for more effective scaffolds that promote robust tissue integration and functional recovery in regenerative medicine applications.

**Figure 3:**
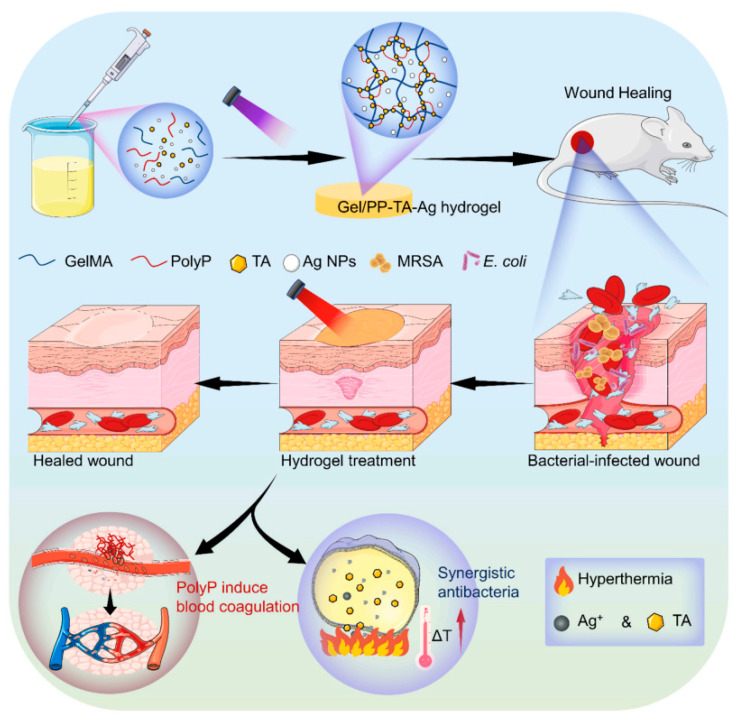
The fabrication process of Gel/PP-TA-Ag hydrogel, designed for wound healing with hemostatic and antibacterial properties. Reprinted with permission from [44]. https://doi.org/10.1016/j.nantod.2021.101165.

**4. Immune Response**: The immunogenicity of hydrogels must be minimized to prevent adverse immune reactions. Strategies to reduce immunogenicity include using naturally derived polymers and modifying synthetic hydrogels with biocompatible coatings [43]. Figure 3 details the step-by-step fabrication process of the Gel/PP-TA-Ag hydrogel, highlighting its integration of components aimed at enhancing wound healing. The hydrogel combines gel matrices with polyphenol-tannic acid (PP-TA) and silver nanoparticles (Ag), which contribute to its dual functionality. The figure illustrates how the inclusion of silver imparts strong antibacterial properties, while the PP-TA component aids in rapid blood clotting, providing effective hemostasis. This multifunctional hydrogel is designed to create an optimal environment for wound healing by preventing infections and promoting tissue regeneration [44].

**5. Degradation Products**: It is essential to ensure that the degradation products of hydrogels are non-toxic and do not accumulate in the body. This can be achieved by careful selection of polymer precursors and cross-linking agents that yield safe byproducts upon degradation [45]. The safety of degradation products is a critical consideration in the design of hydrogels for biomedical applications, particularly those intended for temporary implants and drug delivery systems. As hydrogels degrade, the byproducts must be non-toxic and ideally capable of being metabolized or excreted by the body without causing harm or inducing inflammatory responses. This necessitates the careful selection of polymer precursors and cross-linking agents that generate safe degradation products. For example, natural polymers such as hyaluronic acid or collagen are inherently biocompatible and degrade into harmless metabolites, making them favorable choices for tissue engineering applications. Conversely, synthetic hydrogels often require more stringent design parameters; the chemical structure of the polymer and the nature of the cross-linking agents can greatly influence the toxicity of the resulting degradation products. Researchers must consider factors such as molecular weight, chemical stability, and solubility of the byproducts when selecting materials. Additionally, advancements in polymer chemistry enable the incorporation of biodegradable linkages that yield benign products upon hydrolysis or enzymatic degradation. These innovations allow for a more tailored approach in hydrogel design, ensuring that degradation rates align with tissue healing timelines while maintaining biocompatibility throughout the degradation process. Furthermore, rigorous in vitro and in vivo evaluations are essential to assess the safety profile of degradation products, as the accumulation of toxic byproducts can lead to adverse biological responses and compromise the overall effectiveness of the hydrogel. As the field continues to evolve, the challenge remains to balance the functional performance of hydrogels with the necessity of ensuring that their degradation products contribute positively to patient outcomes and do not hinder the healing process.

## 3. Hydrogels in Tissue Engineering

### 3.1. Scaffold Design and Fabrication

#### 3.1.1. Three-Dimensional Printing Techniques

3D printing, or additive manufacturing, has revolutionized the field of tissue engineering by enabling the precise fabrication of complex scaffold structures. This technique involves layer-by-layer deposition of materials to create three-dimensional constructs that mimic the architecture of natural tissues.

**Figure 4:**
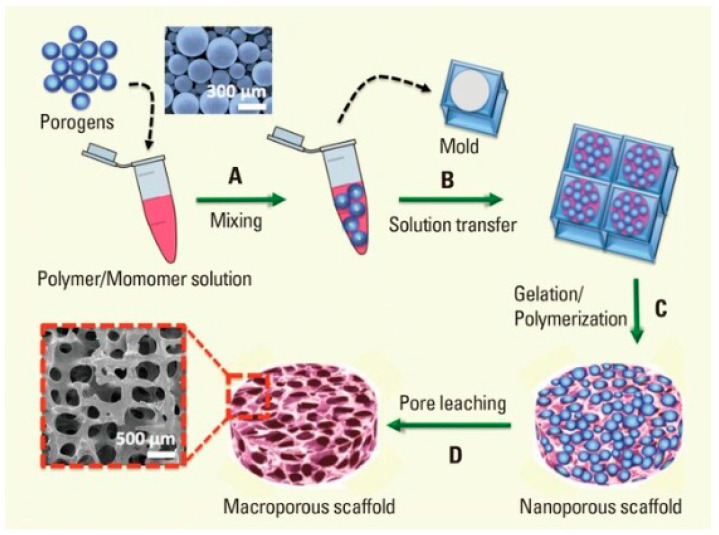
Diagram showing the steps involved in creating macroporous hydrogel scaffolds using sacrificial porogens. (A) Degradable particles are gathered and dispersed in a cross-linkable prepolymer solution. (B) The mixture, containing the porogens (in this case, microspheres), is poured into a mold before solidification. (C) Gelation occurs as the polymerization process encapsulates the leachable porogens. (D) Controlled degradation of the porogens results in the formation of a three-dimensional porous network with interconnected macropores and channels. Reprinted with permission from [47]. https://doi.org/10.5051/jpis.2013.43.6.251.

**1. Stereolithography (SLA)**: SLA is one of the most widely used 3D printing techniques for hydrogel scaffolds. It uses a laser to cure photopolymerizable materials layer by layer. This method allows for high-resolution fabrication, making it suitable for creating intricate microarchitectures. SLA has been used to fabricate scaffolds for bone tissue engineering, where high precision is crucial for mimicking the trabecular bone structure [46]. Figure 4 illustrates the process of fabricating macroporous hydrogel scaffolds using sacrificial porogens. Initially, degradable particles are mixed into a cross-linkable prepolymer solution, creating a suspension. This mixture is then poured into a mold, where the polymerization process encapsulates the porogens as the hydrogel solidifies. The porogens are subsequently removed through controlled degradation, resulting in a porous hydrogel structure with interconnected macropores and channels. This porous architecture is critical for applications requiring enhanced mass transport and cell infiltration, such as tissue engineering [47].

**2. Fused Deposition Modeling (FDM)**: Fused Deposition Modeling (FDM) is a widely utilized 3D printing technique that has recently been adapted for use with hydrogels, enhancing its relevance in tissue engineering and regenerative medicine. Traditionally associated with the extrusion of thermoplastic polymers, FDM’s principles can be effectively applied to hydrogel materials by modifying the nozzle design and temperature parameters to accommodate the unique rheological properties of hydrogels [48]. This adaptation enables the precise deposition of bioinks that retain their integrity during the printing process, allowing for the fabrication of complex scaffold geometries that are crucial for replicating the intricate architecture of native tissues. One of the primary advantages of FDM in hydrogel applications is its capability to incorporate multiple materials within a single scaffold, facilitating the creation of gradient structures that mimic the heterogeneity of biological tissues. Such gradient scaffolds can enhance cell behavior and tissue integration by providing a spatially controlled environment that supports different cell types or functional domains, reflecting the natural composition of tissues like bone or cartilage. Moreover, the ability to adjust parameters such as extrusion speed, layer thickness, and temperature allows for fine-tuning of the mechanical properties and porosity of the printed constructs, further enhancing their suitability for specific applications. However, challenges remain, particularly regarding the optimization of printing conditions to maintain the bioactivity of embedded cells and growth factors, as well as ensuring the fidelity of printed structures post-extrusion. Ongoing research is focused on improving the versatility of FDM by exploring the use of composite hydrogels and developing new formulations that enhance printability while preserving the essential biological functions needed for successful tissue engineering applications. As the technology advances, FDM stands to play a pivotal role in the future of personalized medicine by enabling the design and fabrication of scaffolds tailored to the unique needs of individual patients.

**3. Inkjet Printing**: This technique uses droplets of hydrogel precursor solutions, which are deposited in a precise manner to form a scaffold. Inkjet printing is advantageous for its ability to print cells and bioactive molecules alongside scaffold materials. It has been used to create skin tissue constructs by printing layers of dermal and epidermal cells within a hydrogel matrix [49]. Inkjet printing is a sophisticated 3D printing technique that employs the precise deposition of microdroplets of hydrogel precursor solutions to construct scaffolds for tissue engineering applications. This method stands out for its capability to integrate living cells and bioactive molecules directly into the printed structures, thereby facilitating the development of functional tissue constructs that closely mimic natural tissue architecture. The versatility of inkjet printing allows for the layering of different cell types and extracellular matrix components, making it particularly suitable for applications such as skin tissue engineering, where the formation of distinct layers—such as dermal and epidermal cells—is crucial for creating functional skin grafts. The ability to print complex architectures in a controlled manner supports cellular organization and interaction, which are vital for tissue maturation and functionality. Additionally, inkjet printing allows for high spatial resolution, enabling the creation of intricate patterns that can promote cell behavior and enhance the mechanical properties of the scaffold. However, several challenges need to be addressed, including the optimization of the hydrogel viscosity and the droplet formation process to ensure cell viability during printing. Moreover, the shear stress experienced by cells during the printing process can lead to cell damage, necessitating the careful selection of printing parameters to minimize stress and maintain cellular functionality. The incorporation of bioactive molecules, such as growth factors or signaling peptides, further enhances the bioactivity of the printed constructs, promoting cell proliferation and differentiation. As research in this area advances, inkjet printing holds significant promise for the development of personalized tissue engineering solutions, enabling the production of tailored scaffolds that can effectively support the regeneration of complex tissues while addressing individual patient needs.

**4. Extrusion-Based Bioprinting**: Extrusion-based bioprinting is a widely used technique in tissue engineering that involves the deposition of continuous filaments of hydrogel bioinks through a nozzle, allowing for precise control over scaffold porosity, architecture, and mechanical properties. This method is particularly advantageous for fabricating complex 3D structures, such as cartilage and bone tissue scaffolds [50]. One of the critical aspects of optimizing extrusion-based bioprinting is the use of templating agents, which improve the printability and structural integrity of the scaffolds.

Templating agents play a crucial role in regulating pore size, mechanical strength, and cell infiltration within the scaffold. By incorporating materials such as sacrificial polymers or particles, the bioprinting process can create more controlled and interconnected porous structures, enhancing nutrient diffusion and tissue integration. For example, templating agents like gelatin or poly(lactic-co-glycolic acid) (PLGA) have been used to improve scaffold porosity and mechanical properties in extrusion-based printing for bone and cartilage applications [27,29]. In their work, Pan et al. (2022) highlight the role of templating agents in optimizing the pore structure of printed scaffolds, allowing for the creation of more mechanically robust constructs without compromising biocompatibility. This is particularly important for bone tissue engineering, where both mechanical strength and osteoinductive properties are necessary for successful scaffold integration [29].

Similarly, Torre et al. demonstrated the successful use of templating agents in bioprinting hydrogels to enhance scaffold geometry and mechanical stability, particularly for patient-specific bone implants. Their study underscores how templating agents can be used to modulate the mechanical properties of hydrogel-based scaffolds to match the requirements of different tissue types [27]. Li et al. further explored how templating agents impact the biodegradation rates of hydrogels in scaffold fabrication. By carefully selecting and controlling the degradation behavior of templating materials, the researchers were able to tailor the scaffold’s degradation to match the tissue healing process, ensuring long-term functionality in tissue regeneration [28].

Liang-Tsai et al. also noted that the incorporation of templating agents such as salt or sugar particles could significantly enhance the scaffold’s porosity, creating a more favorable environment for cell infiltration and vascularization during tissue regeneration [31].

Finally, Imani et al. reported on the use of thermosensitive templating agents, which facilitated the removal of sacrificial templates after bioprinting, allowing for the creation of precise and reproducible scaffold architectures with complex geometries. Their study showed that by using templating agents in the extrusion-based bioprinting process, it is possible to significantly improve the printability and functional outcomes of the bio-printed constructs [30]. Thus, templating agents are essential for enhancing the structural and functional properties of bioprinted scaffolds in extrusion-based processes, allowing for more efficient creation of complex, high-precision tissue scaffolds for applications in bone and cartilage tissue engineering.

**5. Digital Light Processing (DLP)**: DLP uses a digital micromirror device to project light patterns onto a photopolymerizable resin, solidifying it layer by layer. This technique is similar to SLA but can achieve faster print speeds due to the parallel projection of light. DLP has been applied in the fabrication of vascularized tissue constructs, where precise control over the scaffold’s internal architecture is essential [51].

The advancement of 3D printing technologies has significantly enhanced the capability to fabricate scaffolds with complex, patient-specific geometries. Moreover, the integration of cells and bioactive molecules during the printing process holds great promise for creating functional tissue constructs that can be used in regenerative medicine [52].

#### 3.1.2. Electrospinning

Electrospinning is a versatile technique for fabricating nanofibrous scaffolds that closely mimic the extracellular matrix (ECM) of natural tissues. The process involves applying a high voltage to a polymer solution or melt, which results in the formation of ultrafine fibers collected on a grounded target.

**1. Fiber Morphology and Alignment**: Fiber morphology and alignment are critical parameters in the electrospinning process, directly influencing the properties and functionality of the resulting scaffolds in tissue engineering applications. By systematically adjusting factors such as polymer concentration, electric field voltage, and the distance between the needle and the collector, researchers can manipulate the diameter, surface morphology, and alignment of the electrospun fibers. Aligned fibers are particularly advantageous for applications targeting anisotropic tissues, such as tendons and nerves, where directional cell growth and tissue organization are essential for functional recovery [53]. Studies have demonstrated that aligned electrospun scaffolds facilitate enhanced cell adhesion, proliferation, and migration along the fiber direction, thereby promoting the formation of aligned extracellular matrix structures that mirror the native tissue architecture. This anisotropic behavior can lead to improved mechanical properties of the scaffold, including tensile strength and elasticity, which are vital for load-bearing applications. Additionally, the fiber diameter can be tuned to influence porosity and surface area, further affecting cellular responses and nutrient diffusion within the scaffold. However, achieving optimal alignment and morphology requires a balance, as excessively high voltages or polymer concentrations can lead to defects in fiber formation or inconsistent alignment. Moreover, post-processing techniques such as mechanical stretching or the incorporation of bioactive molecules may enhance the functional properties of the scaffolds, promoting interactions between cells and the scaffold while supporting tissue integration. Overall, the ability to finely control fiber morphology and alignment through electrospinning provides a powerful tool for designing scaffolds that can effectively mimic the structural and functional properties of native tissues, thereby advancing the field of regenerative medicine.

**2. Material Selection**: Both natural and synthetic polymers can be used for electrospinning. Natural polymers like collagen, gelatin, and silk fibroin provide excellent biocompatibility and promote cell attachment and proliferation. Synthetic polymers such as polycaprolactone (PCL) and polylactic acid (PLA) offer tunable mechanical properties and degradation rates. Blending natural and synthetic polymers can combine the advantages of both, resulting in scaffolds with improved functionality [54,55]. In the realm of cutting-edge hydrogel technologies for tissue engineering and biosensing, selecting appropriate materials presents several challenges, both in terms of the variety of options available and the specific requirements of target applications. Hydrogels are prized for their ability to mimic the extracellular matrix (ECM), support cell proliferation, and facilitate tissue regeneration. However, choosing the ideal hydrogel formulation for specific tissue types or biosensing applications necessitates a careful balance of mechanical properties, biocompatibility, degradability, and functionalization.

**Challenges in Material Selection**

One of the primary challenges in hydrogel-based tissue engineering is the diversity of materials, both natural and synthetic, each offering distinct advantages and drawbacks. Natural hydrogels, such as collagen, alginate, and chitosan, closely resemble biological tissues and are generally biocompatible. However, these materials often suffer from poor mechanical strength and unpredictable degradation rates, which can limit their application in load-bearing tissues like bone or cartilage. Conversely, synthetic hydrogels such as polyethylene glycol (PEG), polyacrylamide (PAAm), and polyvinyl alcohol (PVA) offer tunable mechanical and chemical properties, but their lack of intrinsic bioactivity can hinder cell interactions and tissue integration. In biosensing applications, hydrogels must be responsive to specific biological or chemical stimuli, such as temperature, pH, or the presence of particular biomolecules. Here, stimuli-responsive hydrogels, like poly(N-isopropylacrylamide) (PNIPAAm), show great promise but present additional challenges, such as complex synthesis processes and potential cytotoxicity. Ensuring that these materials remain stable and functional over time while avoiding adverse immune reactions remains a critical challenge.

**Current Uses of Hydrogels**

Hydrogels are currently employed in various tissue engineering applications, from soft tissue regeneration (e.g., skin, cartilage) to more structurally demanding uses like bone tissue engineering. For instance, alginate and collagen are widely used in wound healing and soft tissue regeneration due to their excellent biocompatibility. In contrast, synthetic polymers like PEG are often used for bone tissue scaffolding, where their mechanical tunability and ease of functionalization are advantageous.

In biosensing, hydrogels serve as key components in platforms designed for the detection of glucose, pH, or specific proteins. Responsive hydrogels that undergo physical or chemical changes upon detection of a target analyte are increasingly used in wearable devices and implantable sensors.

Guidelines for Material Selection in Target Applications

To guide material selection, the following factors must be considered:

**Biocompatibility:** The hydrogel material must be non-toxic and elicit minimal immune response. Natural hydrogels like collagen are favored for applications that require close interaction with living cells. Synthetic materials like PEG can be functionalized to improve biocompatibility, but their base forms may require careful assessment of any potential cytotoxicity.

**Mechanical Properties:** Different tissues have vastly different mechanical demands. For example, soft tissues like skin require flexible, highly elastic hydrogels, whereas bone regeneration requires materials with high compressive strength. Therefore, synthetic polymers such as PEG or hybrid composites combining both natural and synthetic hydrogels are preferred for more mechanically demanding applications.

**Degradability:** The rate of hydrogel degradation should align with the rate of tissue regeneration. Hydrogels intended for short-term applications (e.g., wound healing) may prioritize fast degradation, while long-term applications (e.g., bone scaffolds) require slower degradation rates. Biodegradable hydrogels such as chitosan are often employed when temporary scaffolding is necessary, while more stable, cross-linked synthetic hydrogels are chosen for long-term uses.

**Functionalization:** Hydrogels for biosensing or drug delivery often require the incorporation of functional groups or nanoparticles to enable specific responses to external stimuli or to improve their mechanical properties. For example, the addition of gold nanoparticles or the functionalization of PEG hydrogels with specific ligands can enhance the performance of biosensors.

**Scalability and Processability:** For clinical applications, the material must be easy to produce on a large scale and adaptable to various fabrication methods, such as extrusion-based bioprinting. Hydrogels like PEG, which can be easily synthesized and processed, are often preferred for such applications, while more complex, multistep synthesis processes may limit the use of some natural hydrogels.

Ultimately, the choice of hydrogel materials for tissue engineering and biosensing requires balancing several factors, including biocompatibility, mechanical strength, degradation rates, and functionalization potential. Future research must focus on developing materials that can better integrate these properties, offering more precise control over scaffold behavior and functionality, while addressing the specific requirements of each target application.

**Figure 5:**
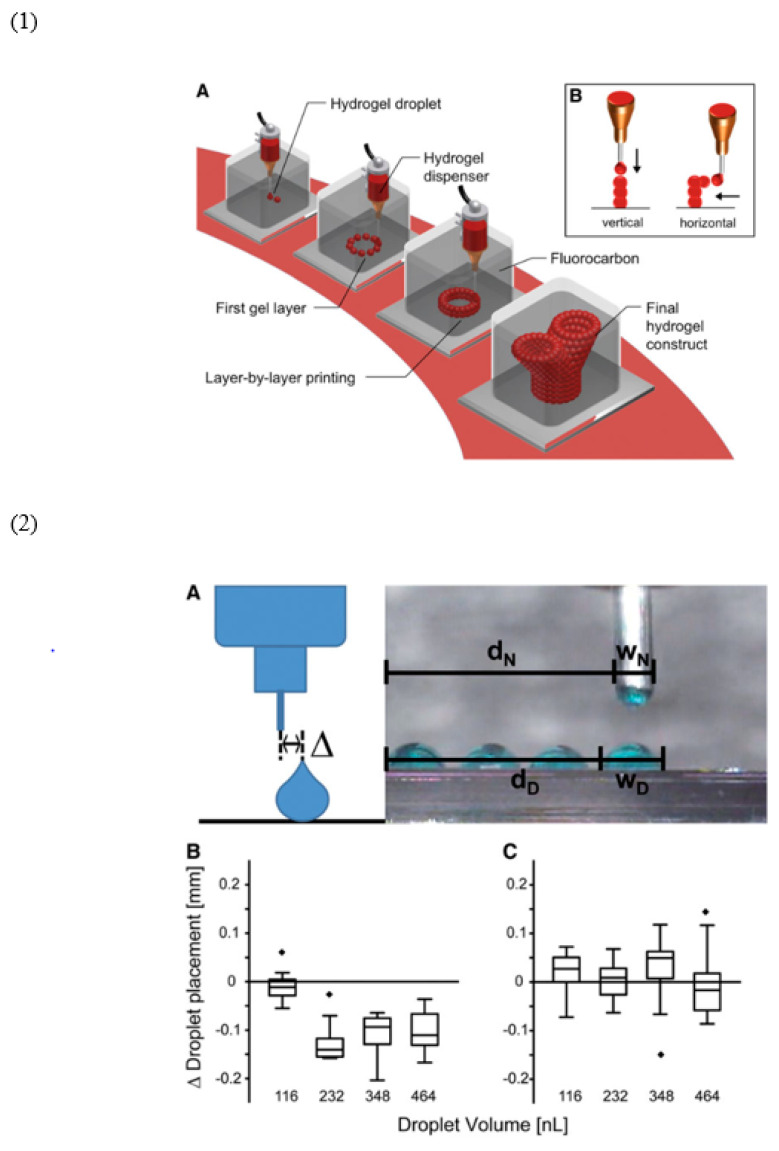
(**1**) Overview of the submerged bioprinting technique. (**A**) Cell-laden hydrogel droplets are sequentially deposited layer by layer following a predefined model to create a three-dimensional tissue structure. This process takes place within a high-density fluorocarbon liquid that provides buoyant support. (**B**) Hydrogel droplets can be added either vertically or horizontally to an existing structure. The fluorocarbon support allows for the creation of branching hydrogel structures or cantilever-like formations without needing a solid foundation. (**2**) Dispensing accuracy. (**A**) To evaluate the precision of the dispensing process, the offset distance (Δ) from the needle’s center to the printed drop was measured, along with the drop diameter. The dispensing deviation was calculated using Equation (1). Printing accuracy was compared between printing in air (**B**) and printing in FC-43 (**C**), with submerged printing showing improved accuracy. The findings are displayed in boxplot diagrams, *n* = 20. Reprinted with permission from [57]. https://doi.org/10.1089/biores.2013.0031.

**3. Functionalization**: Electrospun scaffolds can be functionalized with bioactive molecules to enhance their biological performance. Growth factors, peptides, and other signaling molecules can be incorporated into the fibers or immobilized on their surface to promote specific cellular responses. For example, electrospun scaffolds functionalized with vascular endothelial growth factor (VEGF) have been used to enhance angiogenesis in tissue-engineered constructs [56]. Figure 5 provides a detailed look at the submerged bioprinting technique, highlighting its ability to precisely create complex three-dimensional tissue structures. In this method, cell-laden hydrogel droplets are carefully deposited layer by layer within a supportive fluorocarbon liquid, enabling the formation of intricate architectures like branching or cantilever structures without requiring a solid base. The figure also compares the dispensing accuracy of this technique in air versus in the fluorocarbon liquid, showing that the submerged environment significantly enhances printing precision. These results underscore the potential of submerged bioprinting for advanced tissue engineering applications [57].

**4. Core–Shell Fibers**: Electrospinning can be adapted to produce core–shell fibers, where a core material is encapsulated within a shell of another material. This technique allows for the controlled release of drugs and growth factors from the scaffold, providing sustained delivery to the surrounding tissue. Core–shell electrospun fibers have been used in wound healing applications to deliver antibacterial agents and promote tissue regeneration [58,59]. Core–shell fibers produced through electrospinning represent a significant advancement in scaffold design for tissue engineering and regenerative medicine, allowing for the encapsulation of a core material within a protective shell. This innovative structure provides a unique platform for controlled release of therapeutic agents, such as drugs or growth factors, enhancing the functionality of the scaffold by enabling sustained and localized delivery to surrounding tissues. The ability to tailor the release profile by selecting appropriate materials for both the core and shell components offers considerable advantages in therapeutic applications. For instance, the core can be engineered to release bioactive molecules at specific rates, while the shell can be designed to protect these agents from degradation or premature release. In wound healing applications, core–shell electrospun fibers have been effectively utilized to deliver antibacterial agents, significantly reducing the risk of infection while simultaneously promoting tissue regeneration. The controlled release mechanism can be fine-tuned to match the healing timeline, thereby optimizing therapeutic efficacy. Additionally, the choice of materials for both the core and shell allows for the incorporation of bioactive cues that can further enhance cell responses, such as promoting cell adhesion, migration, and proliferation. The design flexibility offered by core–shell structures also facilitates the integration of multiple functionalities, making it possible to address various aspects of tissue healing in a single scaffold. However, challenges such as the stability of the core–shell structure during processing and the need for precise control over the electrospinning parameters remain areas of active research. By addressing these challenges, core–shell electrospun fibers hold great potential for advancing the field of tissue engineering, particularly in developing multifunctional scaffolds that can enhance patient outcomes through improved therapeutic delivery and tissue integration.

**5. Bioprinting Integration**: Combining electrospinning with 3D bioprinting techniques enables the fabrication of composite scaffolds with enhanced structural and functional properties. Electrospun fibers can be incorporated into 3D-printed constructs to provide additional mechanical strength and a more biomimetic environment for cell growth. This hybrid approach has shown promise in the development of complex tissue-engineered constructs, such as vascularized bone scaffolds [60].

Electrospinning offers significant advantages in terms of scalability and the ability to produce nanofibrous structures that closely resemble the ECM. The ability to functionalize fibers and incorporate bioactive molecules further enhances the potential of electrospun scaffolds in tissue engineering and regenerative medicine [61].

#### 3.1.3. Self-Assembly Methods

Self-assembly methods leverage the intrinsic properties of molecules to form well-defined structures through non-covalent interactions such as hydrogen bonding, hydrophobic interactions, and electrostatic forces. These methods are particularly useful for creating nanostructured scaffolds with precise control over their architecture and functionality.

**1. Peptide-Based Hydrogels**: Peptides can be designed to self-assemble into hydrogels through specific sequences that promote intermolecular interactions. These hydrogels are highly biocompatible and can be tailored to support various cell types. For example, peptide amphiphiles have been developed that self-assemble into nanofibers and support neural cell growth, showing potential for neural tissue engineering [62]. Peptide-based hydrogels have emerged as a promising class of biomaterials for tissue engineering, primarily due to their ability to self-assemble into well-defined structures through specific peptide sequences that promote intermolecular interactions. These hydrogels are highly biocompatible, offering an ideal environment for supporting the growth and differentiation of various cell types, including neural cells. The self-assembly process is driven by non-covalent interactions, such as hydrogen bonding, hydrophobic interactions, and van der Waals forces, which enable the formation of nanostructures like nanofibers, mimicking the extracellular matrix (ECM). Peptide amphiphiles (PAs) represent a notable example, as they consist of a hydrophobic tail and a peptide head that facilitates self-assembly into nanofibrous hydrogels. These nanofibers exhibit structural and functional similarities to natural ECM, making them suitable for applications in neural tissue engineering, where they provide a supportive scaffold for cell adhesion, migration, and differentiation. In addition to their biocompatibility, peptide-based hydrogels can be designed to include specific bioactive motifs that further enhance cellular interactions and guide tissue regeneration. For instance, incorporating sequences that promote neural cell adhesion or stimulate neurite outgrowth has shown great potential in promoting the regeneration of damaged neural tissues. Moreover, the tunable mechanical properties of peptide hydrogels, achieved by modifying peptide sequences or concentrations, allow for the precise control of scaffold stiffness, which is crucial for mimicking the mechanical properties of target tissues. Despite these advantages, challenges remain in optimizing the stability and degradation rates of peptide hydrogels for long-term in vivo applications. Nonetheless, peptide-based hydrogels offer a versatile platform for the development of next-generation biomaterials aimed at advancing tissue engineering and regenerative medicine.

**2. DNA Nanostructures**: DNA-based scaffolds utilize the programmable nature of DNA molecules to form precise nanostructures through base-pairing interactions. DNA hydrogels can be engineered to respond to specific stimuli, such as the presence of enzymes or changes in pH, making them useful for biosensing applications. DNA nanostructures have also been explored for their potential to deliver drugs and genes in a controlled manner [63]. DNA nanostructures represent a cutting-edge approach in biomaterials for tissue engineering and biosensing, leveraging the inherent programmability of DNA molecules to create precisely defined nanostructures through the specificity of base-pairing interactions. These DNA-based hydrogels exhibit unique properties that allow them to be engineered for responsiveness to various stimuli, such as enzymes or pH changes, which enhances their functionality in biosensing applications. For instance, by incorporating specific recognition sequences, these hydrogels can undergo conformational changes in response to target analytes, enabling real-time detection of biomolecules, such as disease markers, with high sensitivity and specificity. Additionally, DNA nanostructures have garnered interest for their potential in controlled drug and gene delivery systems. The ability to design DNA scaffolds that can encapsulate therapeutic agents and release them in response to biological triggers presents significant advantages in achieving localized and sustained delivery, minimizing systemic side effects. Furthermore, the biocompatibility and biodegradability of DNA make it an attractive option for applications involving direct interaction with biological systems. The tunability of DNA nanostructures allows for the customization of their mechanical properties, pore sizes, and degradation rates, facilitating their adaptation for various tissue engineering applications. However, challenges remain regarding the scalability of DNA scaffold production and the stability of DNA in physiological environments, which require ongoing research and optimization. Overall, DNA nanostructures are poised to play a transformative role in the development of next-generation biomaterials, combining precision engineering with biological functionality to advance therapeutic and diagnostic applications.

**3. Block Copolymers**: Block copolymers are versatile biomaterials that can self-assemble into a range of nanostructures, including micelles, vesicles, and nanofibers, dictated by their chemical composition and environmental conditions. This unique ability to form diverse structures enables the tailoring of hydrogels for specific applications in drug delivery and tissue engineering [64]. By adjusting the ratios of hydrophilic and hydrophobic segments within the block copolymers, researchers can manipulate the self-assembly process to produce hydrogels with desired mechanical properties, porosity, and degradation rates. Importantly, these materials can be engineered to degrade under specific conditions—such as pH or temperature changes—facilitating the controlled release of encapsulated therapeutic agents. This attribute is particularly advantageous in drug delivery systems, where maintaining optimal drug concentration at the target site is crucial for therapeutic efficacy while minimizing side effects. In tissue engineering, block copolymer-based hydrogels serve as scaffolds that mimic the extracellular matrix, promoting cell adhesion, proliferation, and differentiation. The tunable mechanical properties of these hydrogels also allow for the design of scaffolds that can match the stiffness of the target tissue, which is essential for successful integration and function. Moreover, the ability to incorporate bioactive molecules within the hydrogel matrix enhances its bioactivity and therapeutic potential. However, challenges such as achieving uniformity in the self-assembled structures and ensuring the stability of the encapsulated agents during the delivery process persist and warrant further investigation. Overall, block copolymer-based hydrogels represent a promising avenue for advancing drug delivery systems and regenerative medicine, offering a platform for the development of sophisticated biomaterials that can respond to physiological conditions while promoting tissue repair and regeneration.

**4. Supramolecular Hydrogels**: Supramolecular hydrogels represent a significant advancement in the field of biomaterials, characterized by their formation through the self-assembly of small molecules driven by non-covalent interactions such as hydrogen bonding, van der Waals forces, and hydrophobic effects. This unique assembly allows for the design of hydrogels that are not only biocompatible but also highly tunable in response to external stimuli, including temperature, light, and pH [65]. Such responsiveness enables the development of dynamic systems capable of adapting to changing environmental conditions, making supramolecular hydrogels particularly appealing for various biomedical applications. For example, in drug delivery, these hydrogels can be engineered to encapsulate anticancer drugs and release them in a controlled manner, responding to specific triggers that enhance the targeting of cancer cells while minimizing systemic exposure. The ability to fine-tune the release kinetics through the selection of appropriate supramolecular interactions ensures that therapeutic agents are delivered precisely when and where they are needed. Additionally, the modular nature of supramolecular assemblies allows for the incorporation of multiple functionalities within a single hydrogel, enabling the simultaneous delivery of drugs and bioactive molecules that can synergistically promote therapeutic outcomes. Despite their advantages, challenges remain regarding the stability and longevity of these hydrogels in physiological environments, as well as the reproducibility of the self-assembly process. Ongoing research is focused on addressing these challenges, enhancing the robustness of supramolecular hydrogels while further exploring their potential in regenerative medicine and targeted drug delivery systems. Overall, the versatility and adaptability of supramolecular hydrogels position them as a promising platform for next-generation biomaterials with a wide array of applications in healthcare.

**5. Protein-Based Hydrogels**: Proteins can self-assemble into hydrogels through specific amino acid sequences that promote intermolecular interactions. Protein-based hydrogels, such as those formed from silk fibroin and elastin-like polypeptides, offer excellent biocompatibility and tunable mechanical properties. These hydrogels have been used to create scaffolds that support cell growth and tissue regeneration in applications ranging from skin to cardiac tissue engineering [66]. Self-assembly methods provide a bottom-up approach to scaffold fabrication, allowing for precise control over the scaffold’s nanostructure and functionality. The versatility of self-assembled scaffolds makes them suitable for a wide range of tissue engineering and drug delivery applications, offering new avenues for creating biomimetic materials that closely replicate the native ECM [67].

### 3.2. Applications in Tissue Regeneration

#### 3.2.1. Bone Tissue Engineering

Bone tissue engineering aims to create constructs that can support bone regeneration and repair, addressing issues such as fractures, bone defects, and degenerative bone diseases. Hydrogels have emerged as promising materials for bone tissue engineering due to their high water content, biocompatibility, and tunable properties.

**1. Biomimetic Scaffolds**: Biomimetic scaffolds are a pivotal advancement in tissue engineering, specifically designed to closely replicate the natural extracellular matrix (ECM) of bone tissue. These hydrogels are engineered to not only mimic the physical architecture of the bone ECM but also to replicate its biochemical cues, which are essential for promoting cellular behaviors critical for bone regeneration. By incorporating bioactive molecules such as growth factors, particularly bone morphogenetic protein-2 (BMP-2), biomimetic hydrogels enhance the osteogenic differentiation of mesenchymal stem cells (MSCs), which are pivotal for bone healing and regeneration. The presence of BMP-2 within the hydrogel matrix not only stimulates MSC proliferation but also facilitates their differentiation into osteoblasts, the cells responsible for bone formation [68]. Furthermore, the structural and compositional fidelity to natural bone ECM supports essential processes like cell attachment and matrix deposition, thereby creating a conducive microenvironment for tissue integration. The tunability of these hydrogels allows for the optimization of mechanical properties and degradation rates to match those of native bone, ensuring that as new tissue forms, the scaffold degrades appropriately without eliciting adverse inflammatory responses. Additionally, advancements in biomimetic designs have led to the incorporation of nanoscale features that further enhance cellular interactions and signaling. Despite the significant progress, challenges remain in terms of achieving the ideal balance between mechanical stability and bioactivity, as well as ensuring that these hydrogels maintain their functional properties in the complex physiological environment of the body. Nonetheless, the development of biomimetic scaffolds stands at the forefront of regenerative medicine, offering promising strategies for effective bone repair and regeneration through the integration of biological and material science.

**2. Composite Hydrogels**: Composite hydrogels that combine organic polymers with inorganic components (e.g., hydroxyapatite, calcium phosphate) are used to enhance the mechanical properties and bioactivity of the scaffolds. These composites support better integration with the native bone and promote mineralization [69]. For example, alginate hydrogels reinforced with hydroxyapatite nanoparticles have shown improved mechanical strength and osteoconductivity. Composite hydrogels represent a significant advancement in the field of biomaterials for bone tissue engineering, combining organic polymers with inorganic components to create scaffolds that exhibit enhanced mechanical properties and bioactivity. The integration of inorganic materials such as hydroxyapatite (HA) or calcium phosphate into the hydrogel matrix not only improves the mechanical strength but also promotes a more favorable interaction with the surrounding biological environment, facilitating better integration with native bone tissue. Hydroxyapatite, in particular, is the primary mineral component of bone, and its incorporation into hydrogels can significantly enhance osteoconductivity, encouraging osteoblast proliferation and mineralization. For instance, alginate hydrogels reinforced with hydroxyapatite nanoparticles have demonstrated not only increased compressive strength but also improved bioactivity, leading to enhanced cell adhesion and proliferation. This synergistic effect enables the scaffolds to mimic the natural bone structure more closely, promoting both mechanical stability and biological functionality. Moreover, the incorporation of HA can also influence the release kinetics of bioactive factors, allowing for sustained delivery that supports tissue regeneration over extended periods. Despite the promising attributes of composite hydrogels, challenges such as achieving uniform dispersion of inorganic particles within the polymer matrix and maintaining the overall biocompatibility of the composite remain critical considerations. Additionally, the interplay between the mechanical properties of the hydrogel and its biological performance must be optimized to ensure effective load-bearing capabilities while supporting cellular activities essential for bone repair. Overall, composite hydrogels are emerging as a versatile platform that leverages the strengths of both organic and inorganic materials to enhance bone regeneration strategies, offering exciting prospects for future developments in regenerative medicine.

**3. Injectable Hydrogels**: Injectable hydrogels offer the advantage of minimally invasive delivery, which is particularly beneficial for irregularly shaped bone defects. These hydrogels can be loaded with cells and bioactive agents and then injected into the defect site, where they gel in situ to form a scaffold that conforms to the defect geometry [70]. Injectable hydrogels based on chitosan and beta-glycerophosphate have been used successfully in bone regeneration studies. Injectable hydrogels have emerged as a promising solution for bone tissue engineering, particularly due to their ability to provide minimally invasive delivery methods for treating irregularly shaped bone defects. The inherent fluidity of these hydrogels allows them to be easily injected into the defect site, where they can gel in situ, conforming to the unique geometry of the defect and thus providing a tailored scaffold for tissue regeneration. This characteristic not only facilitates a better fit but also minimizes surgical trauma, promoting faster recovery. Hydrogels composed of biocompatible materials, such as chitosan combined with beta-glycerophosphate, have shown considerable success in preclinical studies aimed at bone regeneration. Chitosan, a natural polymer known for its biocompatibility and biodegradability, can support cell attachment and proliferation, while beta-glycerophosphate serves as a cross-linking agent that enhances the gelation properties of the hydrogel. The incorporation of bioactive agents, such as growth factors or stem cells, further enhances the therapeutic potential of injectable hydrogels, allowing for localized delivery of these agents directly to the site of injury. The ability to modulate the mechanical properties and degradation rates of these hydrogels also adds to their versatility, making them suitable for various stages of tissue healing. However, challenges remain, such as ensuring adequate gel strength to withstand physiological loads and maintaining the viability of encapsulated cells during the injection process. Additionally, careful consideration must be given to the injectability of the hydrogel, as high viscosity can complicate delivery through standard syringes. Overall, injectable hydrogels represent a significant advancement in bone tissue engineering, offering innovative strategies to enhance the repair and regeneration of bone defects while addressing the practical challenges associated with traditional surgical approaches.

**4. Bioprinting Approaches**: Three-dimensional bioprinting has revolutionized bone tissue engineering by enabling the precise fabrication of scaffolds with controlled architecture using hydrogels as bioinks [71]. Key techniques include extrusion-based bioprinting, which supports high cell density and complex geometries but faces challenges in maintaining cell viability and consistent viscosity [28,29]. Inkjet-based bioprinting provides high resolution for intricate details but is limited by scalability and cell encapsulation issues [27,31]. Laser-assisted bioprinting offers superior spatial resolution and precise cell placement, though it involves complex setups and higher costs [28,30]. Hydrogels like alginate are praised for their biocompatibility and ease of cross-linking, though their mechanical strength can be a limitation for larger constructs [28]. Collagen hydrogels closely mimic the extracellular matrix, enhancing cell adhesion and proliferation, but are expensive and variable [28]. Gelatin methacrylate (GelMA) hydrogels allow for precise control over scaffold properties through photopolymerization, but the process is complex and UV-dependent [29,31]. Polyethylene glycol (PEG)-based hydrogels offer customizable properties and functional groups for improved cell interactions but may lack biological mimicry [29]. The selection of bioprinting techniques and hydrogel materials is critical for developing patient-specific implants that accurately match defect sites, thereby enhancing tissue regeneration [30].

**5. Stimuli-Responsive Hydrogels**: Stimuli-responsive hydrogels that change their properties in response to environmental cues (e.g., pH, temperature, ionic strength) can be used to deliver drugs or growth factors in a controlled manner. These hydrogels can release osteoinductive agents in response to specific triggers, enhancing the bone regeneration process [72]. For instance, pH-sensitive hydrogels can release growth factors in the acidic environment of bone defects. Overall, hydrogels offer a versatile platform for bone tissue engineering, providing customizable properties to support bone regeneration and integration with the host tissue [73]. 

#### 3.2.2. Cartilage Tissue Engineering

Cartilage tissue engineering focuses on regenerating cartilage, a tissue with limited self-repair capacity, to treat conditions such as osteoarthritis and cartilage injuries. Hydrogels are highly suitable for cartilage regeneration due to their hydrophilic nature, which mimics the high water content of native cartilage.

**Figure 6:**
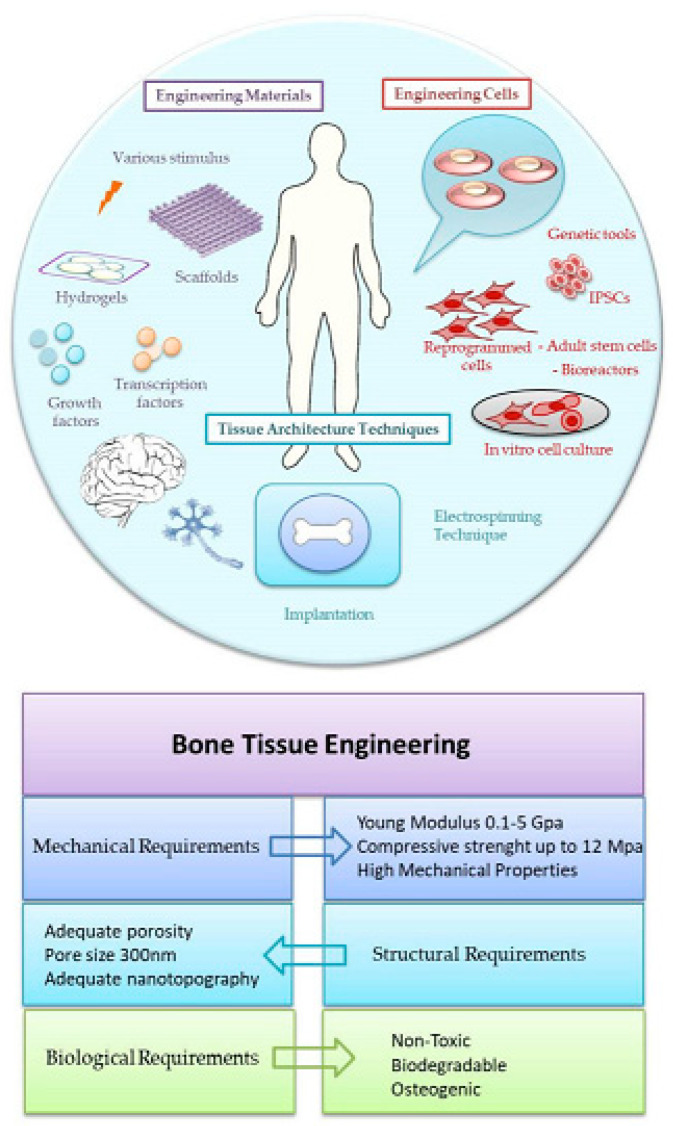
Techniques for designing material, cell, and tissue architectures aimed at tissue engineering applications. Key scaffold requirements for bone tissue engineering are also highlighted. Reprinted with permission from [75]. https://doi.org/10.3390/ma14226899.

**1. ECM-Mimetic Hydrogels**: Hydrogels that mimic the ECM of cartilage can provide a supportive environment for chondrocytes and MSCs. These hydrogels often contain components such as collagen, hyaluronic acid, and chondroitin sulfate to enhance cell adhesion and proliferation [74]. Such hydrogels promote the production of cartilage-specific ECM components, improving the functionality of the engineered cartilage. Figure 6 illustrates various techniques used to design material, cell, and tissue architectures tailored for tissue engineering applications, with a particular focus on bone tissue engineering. The figure emphasizes the importance of scaffold design, highlighting key requirements such as biocompatibility, mechanical strength, and the ability to support cell attachment and growth. These scaffolds play a crucial role in mimicking the natural bone environment, thereby facilitating tissue regeneration. The detailed representation in the figure underscores the complexity and precision required in scaffold design to meet the specific demands of bone tissue engineering [75].

**2. Photopolymerizable Hydrogels**: Photopolymerizable hydrogels represent a versatile and precise approach to hydrogel synthesis, particularly advantageous for tissue engineering applications requiring complex geometries and fine structural control. The cross-linking process is initiated by exposure to light, which allows for spatially regulated polymerization and tuning of mechanical properties. This technique has proven especially effective in developing cartilage constructs, where high resolution and mechanical integrity are critical [76]. For instance, photopolymerizable polyethylene glycol (PEG) hydrogels have been utilized to encapsulate chondrocytes and mesenchymal stem cells (MSCs), facilitating the formation of cartilage tissue. The ability to control scaffold architecture through light exposure enhances cell distribution and promotes uniform tissue regeneration, making this approach highly beneficial for cartilage repair and regeneration. However, challenges such as the potential toxicity of photoinitiators and the need for specific light wavelengths must be carefully managed to ensure biocompatibility and optimal cell viability.

**3. Injectable Hydrogels**: Injectable hydrogels offer significant advantages for cartilage repair, primarily due to their minimally invasive application and ability to conform to irregularly shaped defects. These hydrogels can be delivered in a liquid state and solidify upon injection, forming a supportive scaffold for cell attachment and extracellular matrix (ECM) deposition [77]. Thermosensitive hydrogels, which undergo gelation at body temperature, are particularly promising as they can transition from a liquid to a solid state in situ, providing structural support to damaged cartilage. Injectable hydrogels composed of biocompatible materials like hyaluronic acid and gelatin have demonstrated potential in preclinical studies, showing enhanced cartilage regeneration and integration. Hyaluronic acid, a natural component of cartilage ECM, promotes cell migration and proliferation, while gelatin provides adhesion sites for cells, facilitating tissue repair. However, challenges such as ensuring consistent gelation, maintaining mechanical integrity, and achieving long-term tissue integration remain key areas of ongoing research.

**4. Composite Hydrogels**: Composite hydrogels, which integrate reinforcing materials like nanofibers or nanoparticles into the hydrogel matrix, offer significant improvements in mechanical properties, making them suitable for load-bearing applications such as cartilage and bone tissue engineering. The incorporation of materials such as silk fibroin nanoparticles into alginate hydrogels has been shown to enhance compressive strength while preserving the hydrogel’s biocompatibility. These composite systems not only improve the structural integrity of the scaffold but also promote biological functions. For example, silk fibroin nanoparticles enhance the mechanical resilience of the hydrogel, supporting chondrogenesis and cartilage tissue regeneration [78]. The synergistic effect of combining natural and synthetic components in composite hydrogels allows for better control over mechanical properties and biological outcomes. However, challenges such as ensuring uniform distribution of the reinforcing agents and optimizing degradation rates to match tissue healing remain crucial in advancing their clinical applications.

**5. Gene Delivery Systems**: Hydrogels represent a versatile platform for gene delivery in cartilage tissue engineering, particularly for enhancing the chondrogenic differentiation of mesenchymal stem cells (MSCs) [79]. By incorporating plasmid DNA or viral vectors encoding chondrogenic factors, such as Sox9 or TGF-β, into the hydrogel matrix, a controlled and sustained release of these genetic materials can be achieved. This delivery system enables localized gene expression, which promotes the differentiation of MSCs into chondrocytes and facilitates extracellular matrix (ECM) production. Hydrogels, being biocompatible and easily tunable, offer an ideal microenvironment for this process, supporting not only cell proliferation and differentiation but also customizable mechanical properties that can mimic the native cartilage. In preclinical models, gene-loaded hydrogels have demonstrated potential in repairing cartilage defects, suggesting that this approach could overcome the limitations of traditional therapies by directly enhancing the regenerative capacity of the cells [80]. However, challenges remain in optimizing the gene delivery system, ensuring effective transfection rates, and achieving the precise release kinetics required for consistent therapeutic outcomes.

#### 3.2.3. Skin and Wound Healing

Skin tissue engineering aims to develop constructs that can facilitate the repair and regeneration of skin, particularly in cases of chronic wounds and burns. Hydrogels are ideal for skin regeneration due to their moisture-retentive properties, which promote a favorable healing environment.

**1. Moisture-Retentive Dressings**: Hydrogels provide a moist environment that is conducive to wound healing, reducing dehydration and protecting the wound from external contaminants. These dressings can absorb exudate while maintaining a moist wound bed, which accelerates healing and reduces pain [81].

**2. Antimicrobial Hydrogels**: Incorporating antimicrobial agents into hydrogels can help prevent infections in chronic wounds. Silver nanoparticles, for example, have been used in hydrogel dressings to provide broad-spectrum antimicrobial activity, reducing the risk of wound infection [82]. Hydrogels containing natural antimicrobial peptides have also shown promise in clinical applications.

**3. Growth Factor Delivery**: Hydrogels can be used to deliver growth factors that promote wound healing, such as epidermal growth factor (EGF) and fibroblast growth factor (FGF). These growth factors can be encapsulated within the hydrogel matrix, providing sustained release and enhancing the healing process [83]. Hydrogels containing FGF-2 have been shown to accelerate the healing of diabetic ulcers. Hydrogels have gained significant attention for their role in the controlled delivery of growth factors, which are crucial for promoting wound healing and tissue regeneration. By encapsulating growth factors such as epidermal growth factor (EGF) and fibroblast growth factor (FGF) within the hydrogel matrix, researchers can create a sustained release system that ensures a prolonged presence of these bioactive molecules at the wound site. This sustained release is particularly beneficial in managing complex wounds, such as diabetic ulcers, where timely and localized delivery of growth factors can significantly enhance the healing process. For instance, studies have demonstrated that hydrogels containing FGF-2 accelerate healing by promoting angiogenesis and enhancing the proliferation and migration of fibroblasts, which are essential for tissue repair. The hydrophilic nature of hydrogels not only provides a suitable environment for cell growth but also aids in maintaining a moist wound environment, further facilitating healing. Additionally, the mechanical properties of the hydrogel can be tailored to mimic the natural extracellular matrix (ECM), supporting cellular activities crucial for tissue regeneration. However, challenges remain in optimizing the release kinetics of the encapsulated growth factors, as excessive or inadequate release can hinder the healing process. Furthermore, ensuring the stability of growth factors within the hydrogel during storage and upon application is essential for maintaining their bioactivity. Overall, the integration of growth factor delivery within hydrogel systems represents a promising strategy to enhance wound healing outcomes, offering a versatile platform for advanced therapeutic applications in regenerative medicine.

**4. Cell-Laden Hydrogels**: Hydrogels can serve as carriers for skin cells, such as keratinocytes and fibroblasts, which are essential for wound healing and skin regeneration. Alginate hydrogels loaded with keratinocytes have demonstrated enhanced healing in burn wound models. Cell-laden hydrogels have emerged as a promising approach to skin regeneration and wound healing due to their ability to provide a three-dimensional matrix that mimics the natural extracellular environment [84]. By encapsulating skin cells such as keratinocytes and fibroblasts, these hydrogels promote cell proliferation, migration, and extracellular matrix (ECM) deposition, which are critical processes for effective wound healing. Alginate hydrogels, in particular, have shown significant potential in this field, as they can be easily modified and loaded with therapeutic agents or cells. In burn wound models, alginate hydrogels loaded with keratinocytes have demonstrated enhanced wound closure, faster re-epithelialization, and improved tissue regeneration. These hydrogels not only provide structural support but also maintain an optimal moist environment conducive to healing. Despite these advantages, challenges such as ensuring long-term cell viability and controlling the degradation rate of the hydrogel remain, making further optimization necessary for clinical applications.

**5. Smart Hydrogels**: Smart hydrogels that respond to environmental stimuli, such as pH and temperature, can provide on-demand drug release and other functionalities. For example, pH-responsive hydrogels can release therapeutic agents in response to the acidic environment of infected wounds, enhancing their efficacy [85]. Temperature-responsive hydrogels, particularly those that gel at body temperature, have proven effective for localized antibiotic delivery in wound healing and skin tissue engineering. These hydrogels undergo a phase transition from liquid to gel upon exposure to physiological temperatures, enabling easy application in liquid form and subsequent gelation at the wound site. This property ensures that antibiotics and other bioactive agents are retained locally, providing sustained release and reducing systemic side effects [86]. Beyond drug delivery, these hydrogels also facilitate moisture retention, a critical factor in wound healing, while offering protection against microbial contamination. In skin tissue engineering, temperature-responsive hydrogels provide a scaffold that supports cellular attachment, proliferation, and ECM deposition, creating an environment conducive to tissue regeneration. However, challenges such as optimizing gelation time, mechanical strength, and degradation rates must be addressed to enhance clinical outcomes.

#### 3.2.4. Neural Tissue Engineering

**Figure 7:**
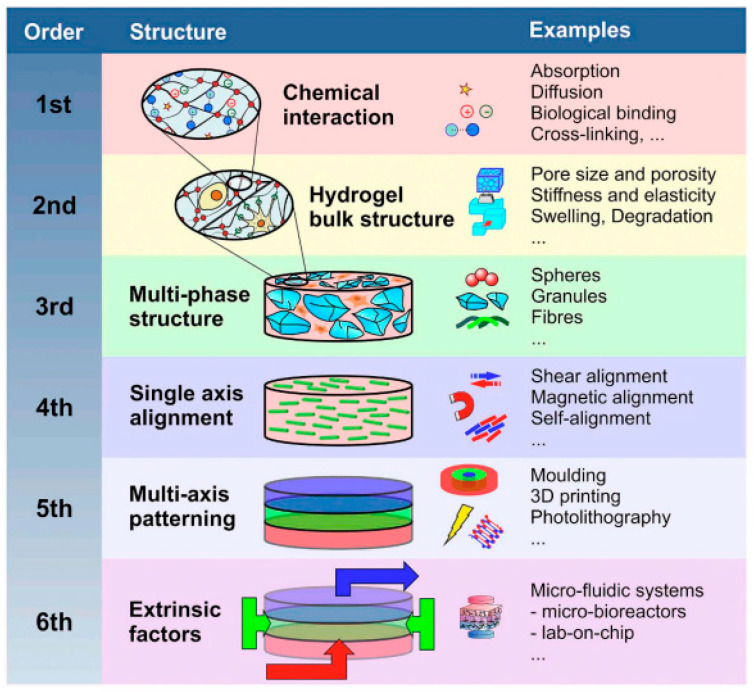
Hierarchical organization of hydrogel structures. The structure of hydrogels can be systematically organized across multiple scales and levels of information content. Starting with material selection, hydrogels can be structured hierarchically from the first to the third order by increasing scale, and from the fourth to the sixth order by adding complexity in information content. The highest level (sixth order) represents a precisely controlled environment, both spatially and temporally, that can mimic the patterning cues observed during neural development. Reprinted with permission from [87]. https://doi.org/10.1016/j.biotechadv.2019.03.009.

Neural tissue engineering seeks to repair or replace damaged neural tissue, addressing conditions such as spinal cord injuries, traumatic brain injuries, and neurodegenerative diseases. Hydrogels are advantageous for neural tissue engineering due to their ability to mimic the soft and hydrated nature of neural tissue. Figure 7 illustrates the hierarchical structuring of hydrogel materials, emphasizing how these structures can be organized across different scales and levels of complexity. The organization begins with the selection of materials, progressing from the first to the third order with increasing physical scale. The fourth to sixth orders introduce higher levels of information content, adding complexity to the hydrogel’s design. At the sixth order, the hydrogel environment becomes highly controlled, capable of replicating the spatial and temporal cues necessary for processes like neural development. This hierarchical approach is crucial for tailoring hydrogels to specific biological applications [87].

**1. Injectable Hydrogels**: Injectable hydrogels provide a minimally invasive approach for delivering cells and therapeutic agents to the injury site. These hydrogels can conform to the complex geometry of neural tissue defects and provide a supportive environment for cell survival and integration [88]. Injectable hyaluronic acid-based hydrogels have been used to deliver neural stem cells to spinal cord injury sites, promoting tissue repair.

**2. Conductive Hydrogels**: Conductive hydrogels represent a significant advancement in neural tissue engineering, as they combine the mechanical properties of traditional hydrogels with enhanced electrical conductivity. By integrating conductive materials like graphene or polypyrrole, these hydrogels facilitate the transmission of electrical signals, which is essential for neuronal communication and overall functional recovery in neural regeneration [89]. The ability of conductive hydrogels to mimic the electrical properties of natural neural tissues allows for improved stimulation of neural cells, thereby enhancing cell survival, proliferation, and differentiation. This feature is particularly advantageous in the context of spinal cord injuries, where conductive hydrogels can act as bridges that not only provide structural support but also restore electrical connectivity across damaged sites. Experimental studies have demonstrated that conductive hydrogels can significantly promote axonal growth and neuronal alignment, leading to improved outcomes in motor and sensory function recovery. Moreover, their tunable mechanical properties enable the customization of stiffness to match that of native neural tissues, thereby enhancing biocompatibility and integration with surrounding tissues. However, challenges remain in ensuring the long-term stability and functionality of the conductive components within the hydrogel matrix, as well as optimizing the balance between mechanical properties and conductivity. Furthermore, the potential cytotoxicity of certain conductive materials must be thoroughly evaluated to ensure safety for in vivo applications. Overall, conductive hydrogels hold great promise as multifunctional scaffolds in neural tissue engineering, paving the way for innovative strategies to promote nerve repair and regeneration.

**3. Neurotrophic Factor Delivery**: Hydrogels have emerged as promising carriers for the delivery of neurotrophic factors, such as nerve growth factor (NGF) and brain-derived neurotrophic factor (BDNF), which play critical roles in neuronal survival, differentiation, and regeneration. The encapsulation of these factors within hydrogel matrices allows for a controlled and sustained release, which is essential for maintaining therapeutic concentrations over extended periods, thereby enhancing neural tissue repair and functional recovery [90]. Research indicates that hydrogels loaded with BDNF significantly promote neuronal differentiation and synaptic connectivity, supporting the formation of functional neural circuits. This sustained release mechanism mimics the natural release patterns of neurotrophic factors in the body, facilitating a more physiological environment that encourages neuronal growth and survival. Additionally, the viscoelastic properties of hydrogels can be tailored to match those of native neural tissues, which further aids in the integration and interaction of transplanted cells or factors with host tissue. Importantly, the use of hydrogels for neurotrophic factor delivery can also mitigate the need for frequent administration, thus improving patient compliance and outcomes. However, challenges remain in optimizing the release kinetics and ensuring the stability of neurotrophic factors within the hydrogel matrix, as they can be sensitive to environmental factors such as temperature and pH. Furthermore, careful consideration must be given to the biocompatibility of the hydrogel materials to prevent adverse inflammatory responses. Overall, the application of hydrogels for neurotrophic factor delivery represents a compelling strategy to enhance neural repair mechanisms, offering exciting possibilities for treating neurodegenerative diseases and injuries.

**4. Three-Dimensional Bioprinting of Neural Constructs**: Three-dimensional bioprinting allows for the fabrication of complex neural tissue constructs with precise spatial control over cell placement and scaffold architecture. Hydrogels serve as versatile bioinks in bioprinting neural tissue constructs, offering a platform that closely mimics the native neural microenvironment. These hydrogels provide the necessary mechanical and biochemical cues that promote cell alignment, network formation, and the development of functional neural circuits. By replicating the anisotropic architecture of neural tissues, hydrogel-based bioinks support the growth and differentiation of neurons, facilitating the formation of synapses and communication pathways critical to neural function [91]. Bioprinted neural constructs are valuable tools for studying neural development and neurodegenerative diseases in vitro, allowing researchers to explore cellular responses, network behaviors, and drug effects in a controlled environment. This approach has the potential to advance personalized medicine and neuroregenerative therapies, though challenges related to replicating the complexity and long-term viability of neural networks remain.

**5. Hydrogel-Nanoparticle Composites**: Incorporating nanoparticles into hydrogels can enhance their mechanical and biological properties, making them more suitable for neural tissue engineering. Gold nanoparticles have emerged as valuable additives to enhance the conductivity and biocompatibility of hydrogels, making them promising candidates for neural tissue engineering applications [92]. These nanocomposites facilitate neuronal differentiation and improve synaptic activity, which is crucial for restoring neural function after injury or disease. By integrating gold nanoparticles into the hydrogel matrix, the electrical properties of the scaffold are significantly enhanced, allowing for better communication between neurons and the scaffold, thereby supporting the development of functional neural networks [93]. Additionally, these nanocomposite hydrogels provide a biocompatible environment conducive to cell survival and integration, promoting neural tissue repair and regeneration. Such multifunctional scaffolds represent an innovative approach in neural tissue engineering, combining mechanical support with bioactivity and electrical conductivity. However, challenges remain in optimizing the long-term stability and fine-tuning the properties of these hydrogels for specific neural regeneration applications, especially in clinical settings.

### 3.3. Hydrogels as Drug Delivery Systems

Hydrogels have garnered significant attention as versatile matrices for drug delivery due to their unique properties such as high water content, biocompatibility, and tunable physicochemical characteristics. These three-dimensional networks of cross-linked hydrophilic polymers can encapsulate drugs and release them in a controlled manner, making them suitable for various biomedical applications.

Hydrogels offer several advantages for drug delivery, including sustained release profiles, protection of drugs from degradation, and the ability to target specific sites within the body [94]. The design of hydrogels involves careful consideration of parameters such as polymer composition, cross-linking density, and environmental responsiveness to achieve desired drug release kinetics [95]. Recent advancements in hydrogel technology have focused on enhancing biocompatibility and biodegradability while improving the loading capacity and release kinetics of therapeutic agents [96]. For instance, novel synthetic polymers or natural polymers modified with functionalities like pH sensitivity or temperature responsiveness have been developed to tailor drug release profiles to specific physiological conditions [97]. Moreover, the integration of nanoparticles or biomolecules into hydrogel matrices has enabled the delivery of bioactive compounds such as proteins, peptides, and nucleic acids with enhanced stability and bioavailability [98]. Ultimate, hydrogels represent a promising platform for drug delivery due to their versatility and ability to address complex therapeutic challenges. Ongoing research continues to explore novel formulations and strategies to optimize hydrogel-based delivery systems for diverse clinical applications.

### 3.4. Challenges and Future Directions

While hydrogels offer significant promise as drug delivery systems, several challenges remain that hinder their widespread clinical translation. Addressing these challenges requires innovative approaches and interdisciplinary collaboration to overcome technical limitations and optimize therapeutic efficacy.

One major challenge is achieving precise control over drug release kinetics from hydrogels [99]. The development of hydrogels with responsive properties (e.g., pH, temperature, enzyme sensitivity) aims to tailor drug release rates to match specific physiological conditions or disease states. However, achieving optimal and reproducible release profiles remains a complex task due to the dynamic nature of biological environments. Another critical issue is ensuring the long-term stability and biocompatibility of hydrogel formulations. The choice of polymers and cross-linking agents significantly impacts the mechanical properties and degradation kinetics of hydrogels in vivo [100]. Advances in material science have led to the development of biodegradable hydrogels that can degrade into non-toxic by-products, minimizing inflammatory responses and promoting tissue regeneration. Furthermore, the scalability and cost-effectiveness of manufacturing hydrogel-based drug delivery systems pose practical challenges [101]. Industrial-scale production methods that maintain the integrity and performance of hydrogels while ensuring batch-to-batch consistency are needed to facilitate commercialization and widespread clinical adoption. Looking forward, future directions in hydrogel-based drug delivery systems include harnessing advanced fabrication techniques such as 3D printing and microfluidics to create customized formulations with spatial and temporal control over drug release [102]. Integration with digital health technologies for real-time monitoring of therapeutic responses and disease progression could further enhance the precision and efficacy of hydrogel-based therapies. So, while challenges exist, ongoing research and technological advancements continue to drive innovation in hydrogel-based drug delivery systems. Collaborative efforts across disciplines are essential to overcome these challenges and unlock the full potential of hydrogels for personalized medicine and targeted drug delivery.

## 4. Hydrogels in Biosensing

### 4.1. Mechanisms of Sensing

#### 4.1.1. Optical Sensors

Optical sensors leverage light to detect and quantify physical properties, chemical compositions, and biological interactions. These sensors are integral to various fields including environmental monitoring, healthcare, and industrial processes due to their high sensitivity, fast response times, and non-invasive nature.

As the working principle of optical sensors operates on principles such as absorption, fluorescence, phosphorescence, and scattering. Typically, an optical sensor system consists of a light source, a medium through which the light passes, and a detector. When the light interacts with the analyte of interest, it undergoes changes in intensity, wavelength, or polarization, which are detected and analyzed.

**1. Absorption-Based Sensors**: Absorption-based sensors are critical tools in various fields, notably in environmental monitoring and chemical analysis, due to their ability to quantify specific substances through changes in light absorption at predetermined wavelengths. The principle underlying these sensors is based on the Beer–Lambert law, which correlates the concentration of a substance in a medium to the amount of light absorbed as it passes through that medium. Non-Dispersive Infrared (NDIR) sensors exemplify this technology, effectively detecting gases like carbon dioxide (CO_2_) by measuring the infrared light absorbed by the gas molecules at characteristic wavelengths [103]. This approach is particularly advantageous for CO_2_ detection because of the gas’s strong absorption features in the infrared spectrum, allowing for highly sensitive and selective measurements. The robustness and reliability of NDIR sensors have made them instrumental in applications ranging from indoor air quality assessments to climate change research. Furthermore, the miniaturization of these sensors has enhanced their applicability in portable devices and real-time monitoring systems. However, challenges persist, including the need for calibration to account for environmental variations and potential interference from other gases. Innovations such as advanced materials for sensor construction and integrated signal processing techniques are actively being explored to improve sensitivity, reduce cross-sensitivity, and enhance overall performance. As the demand for accurate environmental monitoring increases, absorption-based sensors will likely play an increasingly vital role in ensuring compliance with regulatory standards and contributing to sustainability efforts.

**2. Fluorescence Sensors**: Fluorescence sensors are powerful analytical tools that leverage the phenomenon of fluorescence, where a substance absorbs light at one wavelength and subsequently emits light at a longer wavelength. This principle is particularly advantageous in biological assays, where sensitivity and specificity are critical for detecting biomolecules such as nucleic acids, proteins, and other cellular components. The utility of fluorescent sensors is exemplified by fluorescent immunoassays, which combine the specificity of antibodies with fluorescent labeling to enable the detection of specific antigens in complex biological samples [104]. These assays allow for high-throughput screening and real-time monitoring of biomolecular interactions, making them invaluable in fields such as diagnostics, drug discovery, and molecular biology. The development of various fluorescent probes, including quantum dots and fluorescent proteins, has further enhanced the capabilities of these sensors by offering distinct spectral properties, allowing for multiplexing—simultaneous detection of multiple targets in a single assay. Despite their numerous advantages, challenges remain, such as photobleaching, where prolonged exposure to excitation light reduces fluorescence intensity, potentially affecting quantification accuracy. Additionally, the complexity of biological samples can introduce background fluorescence that complicates analysis. To address these issues, researchers are exploring novel strategies such as the use of advanced signal amplification techniques and the development of more stable fluorescent dyes. As the demand for sensitive and rapid detection methods continues to grow, fluorescence sensors are poised to play an increasingly crucial role in advancing biomedical research and clinical diagnostics.

**3. Surface Plasmon Resonance (SPR) Sensors**: Surface Plasmon Resonance (SPR) sensors represent a transformative technology in the realm of biosensing, particularly for real-time analysis of biomolecular interactions. Operating on the principle of surface plasmon waves—coherent oscillations of free electrons at a metal–dielectric interface—SPR sensors detect changes in the refractive index near the sensor surface as biomolecules bind to their targets. This capability allows for the monitoring of binding kinetics and affinity without the need for labeling, which can alter the behavior of biomolecules and introduce artifacts [105]. The real-time, label-free nature of SPR makes it particularly valuable in drug discovery, where understanding the interactions between drug candidates and their targets is crucial. SPR can provide detailed insights into kinetic parameters such as association and dissociation rates, allowing researchers to optimize lead compounds early in the drug development process. Moreover, the versatility of SPR extends to various formats, including parallel assays for high-throughput screening, which enhances its applicability in both academic research and pharmaceutical industries. However, there are challenges to consider, such as the necessity for precise sensor surface functionalization to ensure specific binding, as well as potential limitations in detecting low-abundance analytes due to signal sensitivity. Advances in nanotechnology, such as the integration of gold nanoparticles, are being explored to enhance the sensitivity and broaden the application scope of SPR sensors. As the demand for rapid and accurate biomolecular interaction analysis continues to rise, SPR technology stands at the forefront of biosensing innovation, promising to deepen our understanding of biochemical processes and streamline drug development workflows.

**4. Fiber Optic Sensors**: These sensors transmit light through optical fibers and are used in harsh environments for structural health monitoring, including in bridges and aircraft. The sensitivity and versatility of fiber optic sensors make them ideal for long-distance monitoring [106]. Recent advancements include the development of nanostructured materials to enhance sensor sensitivity and selectivity. Plasmonic nanoparticles, quantum dots, and graphene-based materials have shown promise in various optical sensing applications due to their unique optical properties [107].

However, challenges such as environmental stability, interference from background signals, and the need for miniaturization and integration with other systems remain. Research is ongoing to address these issues, with a focus on developing robust, portable, and cost-effective optical sensors.

#### 4.1.2. Electrochemical Sensors 

Electrochemical sensors are devices that convert chemical information into an electrical signal. They are fundamental in medical diagnostics, environmental monitoring, and industrial process control due to their high sensitivity, specificity, and low cost.

**Figure 8:**
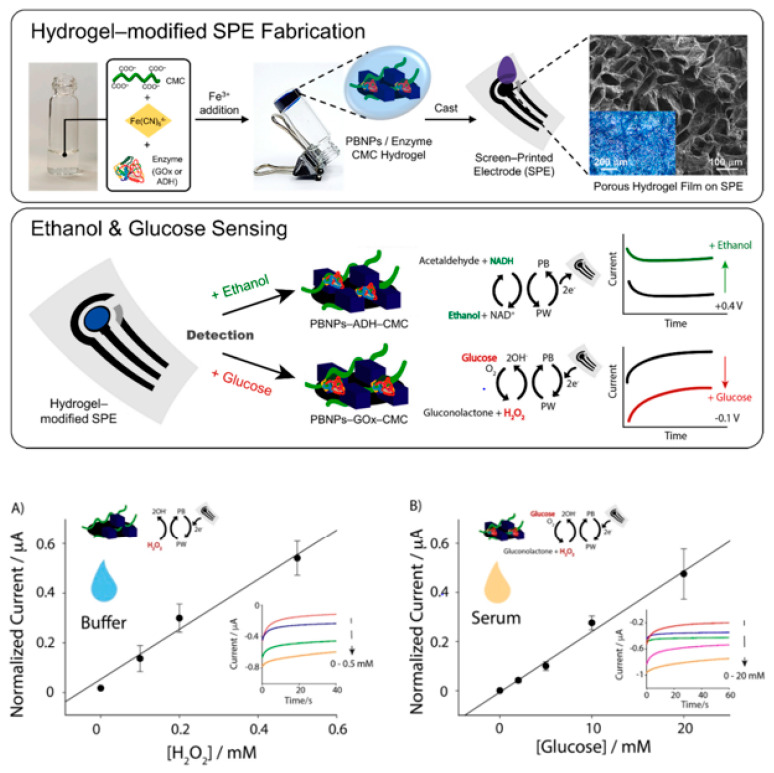
(**Top**) Diagram showing the preparation of hydrogel composites through the in situ synthesis of PBNPs within the CMC matrix in the presence of enzymes (Gox or ADH). The successful formation of the hydrogel composite is demonstrated by a tube inversion test (central image). Modifying flexible screen-printed electrodes (SPE) with the hydrogel composite produces a porous film on the electrode surface, confirmed by SEM and optical microscopy images (inset) of the PBNPs-CMC hydrogel. (**Bottom**) The operational principle of the ethanol biosensor relies on the electrocatalytic oxidation of NADH, generated by ADH-mediated ethanol oxidation on the PBNPs-ADH-CMC modified SPE, while the glucose biosensor is based on the electrocatalytic reduction of H2O2, produced by Gox-mediated glucose oxidation on the PBNPs-Gox-CMC modified SPE. (**A**) Calibration curve for increasing hydrogen peroxide concentrations on PBNPs-CMC modified SPE in 0.05 M phosphate buffer (pH 7.4) with 150 mM KCl. Inset: chronoamperometric curves at −0.1 V (vs. Ag/AgCl). (**B**) Calibration curve for increasing glucose concentrations on PBNPs-Gox-CMC modified SPE in human serum samples. Inset: chronoamperometric curves at −0.1 V (vs. Ag/AgCl). Reprinted with permission from [108]. https://doi.org/10.1016/j.snb.2022.132985.

The working principle of electrochemical sensors functions based on electrochemical reactions occurring at the sensor surface. The key components include a working electrode, a reference electrode, and, in some cases, a counter electrode. When the target analyte interacts with the working electrode, it undergoes oxidation or reduction, producing an electrical signal proportional to its concentration. Figure 8 illustrates the preparation and application of hydrogel composites synthesized in situ with Prussian Blue nanoparticles (PBNPs) within a carboxymethyl cellulose (CMC) matrix in the presence of enzymes like glucose oxidase (Gox) or alcohol dehydrogenase (ADH). The top portion of the figure demonstrates the successful formation of the hydrogel composite, confirmed through a tube inversion test and microscopic analysis, which shows a porous film on modified screen-printed electrodes (SPE). The bottom part of the figure details the operational principles of biosensors created using these composites, highlighting the electrocatalytic processes for detecting ethanol and glucose. The calibration curves for hydrogen peroxide and glucose detection further validate the sensor’s functionality, demonstrating its potential for bioanalytical applications [108].

**1. Potentiometric Sensors**: These measure the potential difference between the working and reference electrodes. Ion-selective electrodes (ISEs), such as pH sensors, are widely used in clinical chemistry and environmental monitoring [109].

**2. Amperometric Sensors**: These measure the current produced by the redox reaction of the analyte. Glucose sensors for diabetes management are a prominent example, where the glucose oxidase enzyme catalyzes the oxidation of glucose, generating a measurable current [110].

**3. Conductometric Sensors**: These measure changes in the electrical conductivity of the sensor material upon interaction with the analyte. They are used for detecting gases such as ammonia and CO_2_ [111].

**4. Impedimetric Sensors**: These measure changes in impedance and are used for detecting biomolecules and pathogens. Electrochemical impedance spectroscopy (EIS) is a powerful technique for characterizing sensor responses [112].

The integration of nanomaterials, such as carbon nanotubes, graphene, and metal nanoparticles, has significantly enhanced the performance of electrochemical sensors by increasing surface area and catalytic activity [113]. Challenges include maintaining the stability and reproducibility of sensors, particularly in complex biological matrices. Efforts are directed toward developing robust sensors with anti-fouling properties and improving selectivity through molecular imprinting and the use of biomimetic materials [114].

#### 4.1.3. Piezoelectric Sensors

Piezoelectric sensors utilize the piezoelectric effect, where mechanical stress generates an electrical charge in certain crystalline materials. These sensors are used in various applications, including pressure sensing, vibration monitoring, and biosensing, due to their high sensitivity and rapid response.

The working principle of the piezoelectric effect occurs in materials such as quartz, lead zirconate titanate (PZT), and polyvinylidene fluoride (PVDF). When subjected to mechanical stress, these materials produce an electric charge proportional to the applied force. The generated electrical signal is then measured and analyzed to determine the magnitude of the mechanical stimulus.

**1. Quartz Crystal Microbalance (QCM)**: QCM sensors measure mass changes on the sensor surface by detecting frequency shifts of a quartz crystal resonator. They are widely used in thin film deposition monitoring and biosensing applications, including DNA hybridization and protein binding studies [115].

**2. Surface Acoustic Wave (SAW) Sensors**: SAW sensors utilize acoustic waves traveling along the surface of a piezoelectric material. They are used for gas detection, pressure sensing, and biosensing due to their high sensitivity to surface perturbations [116].

**3. Bulk Acoustic Wave (BAW) Sensors**: BAW sensors measure changes in bulk properties of the piezoelectric material. They are used in applications such as pressure sensing and as frequency control devices in communication systems [117].

Recent advancements in piezoelectric sensors include the development of flexible and wearable sensors using piezoelectric polymers and composites. These sensors are finding applications in healthcare monitoring, including wearable devices for continuous health tracking [118]. Challenges in piezoelectric sensing include maintaining sensor stability and performance under varying environmental conditions. Research is focused on developing robust materials and improving the sensitivity and selectivity of piezoelectric sensors through material engineering and the integration of advanced signal processing techniques [119]. So, optical, electrochemical, and piezoelectric sensors represent critical technologies for sensing a wide range of physical, chemical, and biological phenomena. Each sensor type offers unique advantages and faces specific challenges. Ongoing research and development are essential to enhance their performance, reliability, and applicability in real-world scenarios. The integration of nanomaterials, advanced fabrication techniques, and signal processing methods holds great promise for the future of these sensors, enabling more accurate, sensitive, and versatile sensing solutions.

### 4.2. Design of Hydrogel-Based Sensors

Hydrogel-based sensors have garnered significant attention due to their unique properties such as high water content, biocompatibility, and tunable mechanical properties. These characteristics make hydrogels ideal for applications in biosensing, environmental monitoring, and healthcare. The design of hydrogel-based sensors involves integrating smart and responsive elements to achieve high sensitivity, specificity, and functionality.

#### 4.2.1. Smart Hydrogels

Smart hydrogels, also known as intelligent or stimuli-responsive hydrogels, can undergo significant changes in their physical or chemical properties in response to external stimuli such as temperature, pH, light, or the presence of specific ions or molecules. These changes make them suitable for a variety of sensing applications.

**1. Temperature-Responsive Hydrogels**: These hydrogels exhibit a phase transition in response to temperature changes. Poly(N-isopropylacrylamide) (PNIPAM) is a widely studied thermoresponsive hydrogel that undergoes a volume phase transition at its lower critical solution temperature (LCST) around 32 °C. Below this temperature, PNIPAM is hydrophilic and swollen, while above it, the hydrogel becomes hydrophobic and collapses. This property is exploited in drug delivery and cell culture applications where a controlled response to temperature is essential [120].

**2. pH-Responsive Hydrogels**: These hydrogels respond to changes in pH by altering their swelling behavior. They are typically composed of polymers with ionizable groups that accept or donate protons in response to pH changes. For example, hydrogels containing polyacrylic acid (PAA) swell in basic conditions due to ionization of carboxyl groups, making them useful for applications such as targeted drug delivery in the gastrointestinal tract [121].

**3. Light-Responsive Hydrogels**: These hydrogels change their properties upon exposure to light, usually through the incorporation of photochromic compounds or nanoparticles that can absorb light and convert it into heat or cause a chemical reaction. Azobenzene derivatives are common photoresponsive components that undergo reversible trans-cis isomerization upon light exposure, leading to changes in the hydrogel’s structure and properties [122].

**4. Ion-Responsive Hydrogels**: These hydrogels swell or contract in response to the presence of specific ions. This response is often due to electrostatic interactions between the hydrogel’s polymer network and the ions. For instance, alginate hydrogels can undergo ionotropic gelation in the presence of calcium ions, making them suitable for applications in wound healing and drug delivery [123].

**1. Biosensing**: Smart hydrogels can be designed to recognize specific biomolecules, making them valuable for biosensing applications. For example, glucose-responsive hydrogels containing glucose oxidase can change their swelling behavior in the presence of glucose, enabling continuous glucose monitoring for diabetes management [124].

**2. Environmental Monitoring**: Hydrogels that respond to environmental stimuli such as pollutants or changes in pH can be used for monitoring water quality. pH-responsive hydrogels embedded with fluorescent dyes can act as pH indicators for real-time monitoring of water bodies [125].

**3. Drug Delivery**: Smart hydrogels are widely used in controlled drug delivery systems. Their ability to release drugs in response to specific stimuli such as temperature or pH ensures targeted and controlled release, improving therapeutic outcomes [126].

Recent advancements in smart hydrogel design include the development of multi-responsive hydrogels that can respond to multiple stimuli simultaneously, offering enhanced functionality and versatility. Additionally, the incorporation of nanomaterials such as graphene and carbon nanotubes has improved the mechanical properties and responsiveness of hydrogels [127].

However, challenges remain in ensuring the long-term stability and biocompatibility of smart hydrogels, particularly for in vivo applications. Research is ongoing to address these issues by developing new polymer compositions and cross-linking methods that enhance the durability and safety of smart hydrogels [128].

#### 4.2.2. Responsive Hydrogels

Responsive hydrogels, a subset of smart hydrogels, are specifically designed to undergo significant changes in response to external stimuli, making them particularly useful in dynamic environments where real-time sensing and adaptation are required.

**1. Chemical-Responsive Hydrogels**: These hydrogels respond to specific chemical stimuli such as the presence of particular ions or molecules. The response is often mediated through specific binding interactions or changes in the chemical environment. For instance, hydrogels containing boronic acid moieties can bind to diols like glucose, resulting in changes in swelling behavior, which is useful for glucose sensing [129].

**2. Biological-Responsive Hydrogels**: Biologically responsive hydrogels represent a cutting-edge approach to drug delivery and therapeutic applications, offering significant advantages by leveraging the body’s own biochemical environment. These hydrogels are engineered to respond to specific biological stimuli, such as enzymes, pH changes, or the presence of particular biomolecules, allowing for precise control over drug release and therapeutic efficacy. For instance, enzyme-responsive hydrogels that degrade in the presence of matrix metalloproteinases (MMPs) have gained prominence in cancer therapy, as MMPs are overexpressed in many tumor microenvironments [129]. This specificity enables targeted drug release directly at the tumor site, minimizing systemic side effects and enhancing localized treatment effectiveness. Additionally, the incorporation of bioactive agents or therapeutic molecules into these hydrogels can be finely tuned to respond to the levels of specific enzymes, allowing for a feedback mechanism where drug release is dictated by the pathological state of the tissue. This dynamic interaction not only improves the therapeutic index but also provides an adaptive approach to treatment, accommodating the variability in tumor biology. However, challenges remain, including the need for precise control over the hydrogel’s degradation kinetics and the potential for off-target effects if enzymes are present in non-target tissues. Ongoing research is focused on optimizing the design of these hydrogels, exploring hybrid systems that combine multiple stimuli-responsiveness, and enhancing the specificity of interactions to further improve their application in personalized medicine and targeted therapies. This innovative approach not only holds promise for improving treatment outcomes but also paves the way for developing smart therapeutics that can adjust their release profiles based on real-time biological feedback.

**3. Magnetic-Responsive Hydrogels**: These hydrogels contain magnetic nanoparticles that respond to external magnetic fields. The magnetic field can induce changes in the hydrogel’s structure, leading to controlled drug release or mechanical actuation. Magnetic-responsive hydrogels are explored for remote-controlled drug delivery systems and soft robotics [130]. Also many other applications like

**Figure 9:**
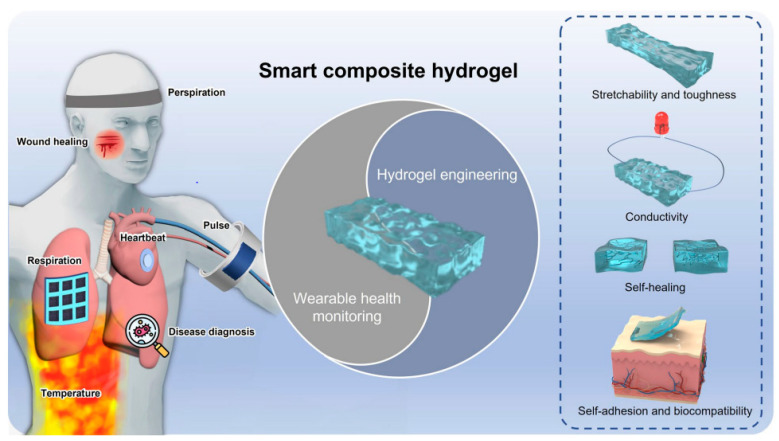
Design and development of advanced composite hydrogels tailored for wearable health monitoring applications. Reprinted with permission from [131]. https://doi.org/10.1007/s40820-023-01079-5.

**a. Medical Diagnostics**: Responsive hydrogels are employed in diagnostic devices for detecting biomarkers. For example, hydrogels that respond to specific proteins or nucleic acids can be used in biosensors to detect diseases at an early stage. These hydrogels can be incorporated into point-of-care diagnostic devices, providing rapid and accurate results [17]. Figure 9 presents the design and development process of advanced composite hydrogels specifically engineered for wearable health monitoring applications. The figure highlights how these hydrogels are tailored to meet the demands of flexibility, biocompatibility, and responsiveness, which are essential for integration with wearable devices. The hydrogels are designed to be sensitive to physiological signals, enabling real-time monitoring of health parameters. The figure also likely demonstrates the layering and functionalization techniques used to enhance the performance of these hydrogels, making them suitable for continuous and non-invasive health monitoring [131].

**b. Drug Delivery Systems**: The ability of responsive hydrogels to release drugs in response to specific stimuli makes them ideal for targeted and controlled drug delivery. Hydrogels that degrade or swell in response to pH changes can be used to deliver drugs to specific parts of the body, such as the stomach or intestines, where the pH environment is different [132].

**c. Tissue Engineering**: Responsive hydrogels are used in tissue engineering to create scaffolds that can adapt to the biological environment. Hydrogels that respond to cellular signals can promote cell growth and differentiation, aiding in tissue regeneration. For example, hydrogels that release growth factors in response to cellular activity can enhance tissue repair processes [133].

Advancements in responsive hydrogel technology include the development of hydrogels with enhanced sensitivity and specificity to stimuli. Researchers are exploring the use of biomimetic materials to create hydrogels that can more accurately replicate natural tissue responses. Additionally, the integration of responsive hydrogels with electronic and microfluidic systems has led to the creation of hybrid devices with improved functionality [134].

Challenges in the field of responsive hydrogels include ensuring their stability and performance over time, particularly in complex biological environments. Ensuring biocompatibility and minimizing potential toxicity are critical for clinical applications. Moreover, scaling up the production of responsive hydrogels while maintaining their functional properties remains a significant hurdle [135]. So the design of hydrogel-based sensors, particularly those utilizing smart and responsive hydrogels, offers immense potential for a wide range of applications in biosensing, environmental monitoring, drug delivery, and tissue engineering. Continued research and development in this field are crucial for overcoming existing challenges and enhancing the performance, stability, and functionality of these innovative materials. The integration of advanced materials and technologies will pave the way for the next generation of hydrogel-based sensors with unprecedented capabilities.

### 4.3. Applications in Medical Diagnostics

**Figure 10:**
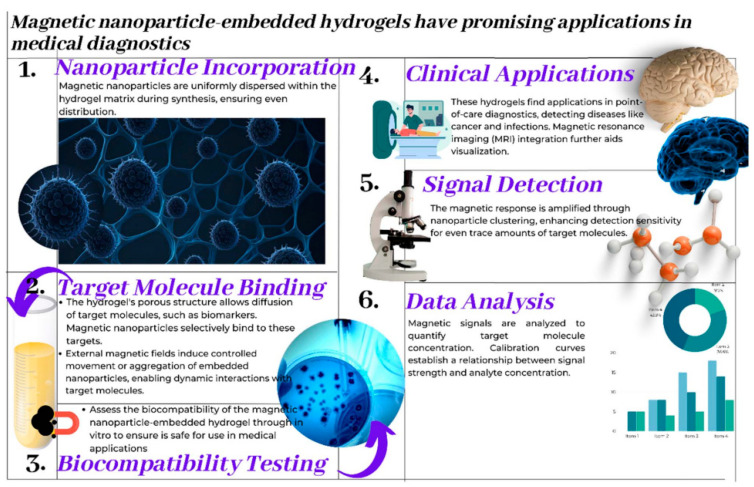
Investigating the function of magnetic nanoparticle-infused hydrogels in diagnostic applications. Reprinted with permission from [96]. https://doi.org/10.1039/D3RA07391B.

Hydrogel-based sensors have found extensive applications in medical diagnostics due to their excellent biocompatibility, tunable properties, and ability to respond to specific biological stimuli. The integration of hydrogels with diagnostic tools enables sensitive and selective detection of various analytes, crucial for disease management and treatment. This section discusses the applications of hydrogel-based sensors in glucose monitoring, pathogen detection, and biomarker detection. Figure 10 explores the role of magnetic nanoparticle-infused hydrogels in diagnostic applications. The figure likely illustrates how these hydrogels are engineered to respond to external magnetic fields, allowing for precise control and manipulation in diagnostic procedures. Magnetic nanoparticles embedded within the hydrogel matrix can enhance imaging contrast or enable targeted delivery of diagnostic agents. Additionally, the figure may depict the integration of these hydrogels into diagnostic devices, highlighting their potential for improving the sensitivity and specificity of various diagnostic tests. The combination of magnetic responsiveness and hydrogel flexibility makes these materials highly versatile for advanced diagnostic applications [96].

#### 4.3.1. Glucose Monitoring

Glucose monitoring is critical for the management of diabetes, a chronic disease affecting millions worldwide. Traditional glucose monitoring methods involve invasive techniques like finger-prick blood tests, which can be uncomfortable and inconvenient for patients. Hydrogel-based sensors offer a promising alternative by providing continuous, non-invasive glucose monitoring.

**1. Glucose Oxidase-Based Hydrogels**: One common approach involves incorporating glucose oxidase (GO_x_) into hydrogels. GO_x_ catalyzes the oxidation of glucose to gluconic acid and hydrogen peroxide, which can be detected electrochemically or optically. Hydrogels containing GO_x_ and electrochemical transducers can monitor glucose levels in real time by measuring the current generated from the reaction [136].

**2. Phenylboronic Acid-Based Hydrogels**: Phenylboronic acid (PBA) is another popular material for glucose sensing. PBA can reversibly bind with glucose to form a cyclic ester, leading to changes in the hydrogel’s swelling behavior. This change can be transduced into an optical signal, such as a shift in fluorescence or color, providing a continuous glucose monitoring platform [137].

Recent advancements in hydrogel-based glucose sensors include the development of wearable devices and implantable sensors. Wearable glucose sensors, often integrated into patches or contact lenses, offer continuous glucose monitoring without the need for blood samples. For instance, hydrogel-based microneedle patches can painlessly penetrate the skin to monitor interstitial glucose levels [138].

However, challenges remain in ensuring the long-term stability and accuracy of these sensors. The biocompatibility of the hydrogels and the prevention of biofouling (the accumulation of proteins and cells on the sensor surface) are critical issues that researchers are addressing through surface modifications and the incorporation of antifouling agents [139].

The future of hydrogel-based glucose monitoring lies in the development of multifunctional sensors that can simultaneously monitor multiple biomarkers and deliver therapeutic agents. Additionally, integrating these sensors with digital health platforms could enhance diabetes management by providing real-time data to patients and healthcare providers, enabling personalized treatment plans [140].

#### 4.3.2. Pathogen Detection

Rapid and accurate detection of pathogens is essential for diagnosing infectious diseases and preventing outbreaks. Hydrogel-based sensors have emerged as powerful tools for pathogen detection due to their high sensitivity, specificity, and ability to operate in complex biological environments.

**1. Nucleic Acid-Based Hydrogels**: Nucleic acid-based hydrogels have emerged as a highly innovative platform for biosensing applications, particularly in the detection of pathogens. These hydrogels are engineered to incorporate nucleic acids, such as DNA or RNA aptamers, which can selectively bind to specific target sequences, including those found in viral or bacterial genomes. The binding event triggers significant changes in the physical properties of the hydrogel, often resulting in a gel-to-sol transition or alterations in optical characteristics, which can be quantitatively monitored. This responsiveness is typically achieved through carefully designed molecular interactions, where the formation of stable duplexes or triplexes between the aptamer and the target nucleic acid destabilizes the hydrogel structure. The use of nucleic acid aptamers offers several advantages, including high specificity and affinity for their targets, along with the potential for multiplexing capabilities, allowing for the simultaneous detection of multiple pathogens [141]. Moreover, the integration of signal amplification strategies, such as the incorporation of fluorescent labels or other signaling moieties, enhances the sensitivity of these hydrogels, making them suitable for early diagnosis in clinical settings. However, challenges remain in optimizing the hydrogel formulation to maintain stability and functionality under physiological conditions, as well as ensuring that the nucleic acid components are resistant to degradation. Research is ongoing to address these issues, exploring various cross-linking techniques and polymer compositions that can improve the robustness and efficacy of nucleic acid-based hydrogels in real-world applications, such as point-of-care diagnostics and environmental monitoring. This approach not only promises rapid and reliable pathogen detection but also reflects a significant advancement in the field of bioengineering, harnessing the specificity of nucleic acids for enhanced biosensing capabilities.

**2. Antibody-Based Hydrogels**: Hydrogels can be incorporated with antibodies that specifically bind to antigens on the surface of pathogens. Upon binding, these hydrogels can generate detectable signals, such as fluorescence or color change, indicating the presence of the pathogen. This approach is widely used for detecting bacteria and viruses, including those responsible for diseases like COVID-19 and influenza [142].

One significant advancement in hydrogel-based pathogen detection is the development of point-of-care (POC) diagnostic devices. These portable and easy-to-use devices allow for rapid on-site detection of pathogens without the need for sophisticated laboratory equipment. For example, lateral flow assays incorporating hydrogel-based detection elements have been developed for rapid COVID-19 testing, providing results within minutes [143]. Challenges in this field include ensuring the specificity of the hydrogel sensors to avoid false positives and negatives. The stability of the biological recognition elements (antibodies, nucleic acids) within the hydrogel matrix over time is another critical issue. Researchers are exploring various stabilization techniques, such as cross-linking and encapsulation, to enhance the longevity of these sensors [144].

The integration of hydrogel-based pathogen sensors with advanced technologies such as microfluidics and artificial intelligence holds great promise. Microfluidic devices can automate and streamline the detection process, while AI algorithms can analyze the sensor data to provide accurate diagnostics and predictive insights. These advancements could revolutionize the field of infectious disease diagnostics by enabling rapid, precise, and scalable pathogen detection [145].

#### 4.3.3. Biomarker Detection

Biomarkers are measurable indicators of physiological and pathological processes, and their detection is crucial for diagnosing diseases, monitoring treatment efficacy, and predicting patient outcomes. Hydrogel-based sensors offer a versatile platform for biomarker detection due to their tunable properties and ability to incorporate various recognition elements.

**1. Enzyme-Based Hydrogels**: Enzymes can be immobilized within hydrogels to catalyze reactions with specific biomarkers, leading to detectable changes. For example, hydrogels containing horseradish peroxidase (HRP) can catalyze the oxidation of substrates in the presence of hydrogen peroxide, producing a color change that indicates the presence of biomarkers like hydrogen peroxide [146].

**2. Aptamer-Based Hydrogels**: Aptamers are short, single-stranded nucleic acids that can bind to specific targets with high affinity. Hydrogels incorporating aptamers can detect a wide range of biomarkers, including proteins, small molecules, and cells. The binding of the target biomarker to the aptamer can induce conformational changes in the hydrogel, producing measurable optical or mechanical signals [147].

One notable advancement is the development of multiplexed hydrogel sensors that can simultaneously detect multiple biomarkers. These sensors are particularly valuable for comprehensive disease diagnostics, allowing for the simultaneous monitoring of several biomarkers associated with a particular condition. For instance, multiplexed hydrogel sensors have been developed for detecting cancer biomarkers, providing valuable information for early diagnosis and treatment monitoring [148].

Challenges in biomarker detection using hydrogel-based sensors include ensuring the sensitivity and specificity of the sensors in complex biological fluids, such as blood or urine. Non-specific binding and interference from other biomolecules can affect the accuracy of the detection. Researchers are addressing these issues by optimizing the hydrogel composition and incorporating antifouling coatings to reduce non-specific interactions [149].

The future of hydrogel-based biomarker detection lies in the development of wearable and implantable sensors that provide continuous monitoring of biomarkers in real time. These devices could revolutionize personalized medicine by enabling continuous health monitoring and early detection of diseases. Additionally, integrating these sensors with wireless communication technologies could allow for remote health monitoring and data sharing with healthcare providers [150].

### 4.4. Environmental and Industrial Applications

Hydrogel-based sensors are increasingly being recognized for their utility in environmental and industrial applications. Their ability to swell or shrink in response to various stimuli, combined with their biocompatibility and versatility, makes them ideal for detecting pollutants and monitoring food quality. This section explores the use of hydrogel-based sensors in pollution detection and food quality monitoring, highlighting recent advancements and challenges.

#### 4.4.1. Pollution Detection

Environmental pollution is a critical global issue, necessitating the development of efficient and sensitive detection methods. Hydrogel-based sensors offer a promising solution for real-time monitoring of various pollutants, including heavy metals, organic contaminants, and gases.

**1. Heavy Metal Detection**: Hydrogels can be functionalized with chelating agents that selectively bind to heavy metals like lead, mercury, and cadmium. These hydrogels change their optical properties (e.g., color, fluorescence) upon metal binding, enabling visual or spectrophotometric detection. For example, hydrogels incorporating thiol groups have shown high sensitivity and selectivity for mercury ions [151].

**Figure 11:**
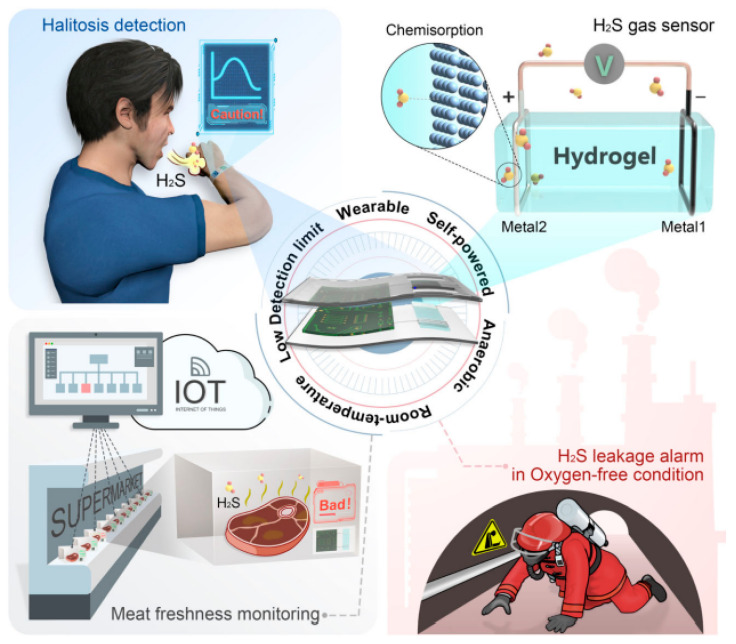
Diagram depicting a flexible gas sensor device and its use in detecting bad breath, monitoring meat freshness, and alerting for hydrogen sulfide leaks. Reprinted with permission from [153]. https://doi.org/10.1038/s41467-023-40953-z.

**2. Organic Contaminant Detection**: Hydrogels containing molecularly imprinted polymers (MIPs) can selectively recognize and bind to specific organic molecules. These hydrogels can detect pollutants such as pesticides, herbicides, and industrial solvents by changing their swelling behavior or generating an electrochemical signal upon binding to the target molecule [152]. Figure 11 illustrates a flexible gas sensor device designed for versatile applications. The diagram showcases the sensor’s ability to detect various gases, such as those related to bad breath, which can be crucial for oral health monitoring. It also highlights its role in assessing meat freshness, an important aspect of food safety. Additionally, the device is equipped to detect hydrogen sulfide leaks, providing critical safety alerts in industrial settings. The flexibility of the sensor device allows for easy integration into different environments and ensures reliable performance across multiple applications [153].

**3. Gas Detection**: Hydrogels can also be used for detecting environmental gases like ammonia, carbon dioxide, and sulfur dioxide. By incorporating pH-sensitive dyes or fluorescent molecules, these hydrogels can provide a visual indication of gas concentration through color changes or fluorescence intensity variations [154].

Recent advancements in hydrogel-based pollution detection include the development of smart materials that can respond to multiple pollutants simultaneously. These multi-responsive hydrogels can provide comprehensive environmental monitoring in real time. Additionally, the integration of hydrogel sensors with wireless and IoT technologies enables remote and continuous monitoring of pollution levels [155].

However, several challenges remain. Ensuring the long-term stability and durability of hydrogel sensors in harsh environmental conditions is critical. Moreover, the selectivity and sensitivity of the sensors must be optimized to avoid interference from other substances present in the environment [156].

Future research should focus on developing self-healing and regenerable hydrogel sensors that can maintain their functionality over extended periods. Enhancing the sensitivity and selectivity of these sensors through advanced materials and nanotechnology will further improve their performance. Additionally, integrating hydrogel sensors with data analytics platforms can provide valuable insights into pollution trends and facilitate more effective environmental management [157].

#### 4.4.2. Food Quality Monitoring

Ensuring food quality and safety is a major concern for consumers, producers, and regulatory bodies. Hydrogel-based sensors can play a significant role in monitoring various parameters related to food quality, such as freshness, contamination, and spoilage.

**1. Freshness Indicators**: Freshness indicators based on hydrogels represent a novel approach to food safety and quality monitoring. By incorporating pH-sensitive dyes or enzymes that respond to the volatile compounds generated during food spoilage, these hydrogels offer a visually intuitive means of assessing food freshness. For example, hydrogels functionalized with bromocresol green exhibit a distinct color change in response to pH fluctuations, which are often linked to the production of organic acids by decaying microorganisms [158]. This transformation occurs due to the hydrogel’s ability to absorb moisture and interact with the acidic byproducts, effectively translating chemical changes into a perceptible visual signal. The integration of such indicators is particularly advantageous as it provides consumers and retailers with immediate feedback on the edibility of food products without the need for complex instrumentation. Additionally, the use of biodegradable and biocompatible materials in hydrogel formulations aligns with growing consumer preferences for environmentally friendly solutions. However, challenges remain in ensuring the specificity and sensitivity of these indicators to avoid false positives or negatives, as well as maintaining the stability of the dye or enzyme within the hydrogel matrix under varying storage conditions. Continued research focuses on optimizing the composition and cross-linking density of these hydrogels to enhance their responsiveness and durability. Overall, the development of freshness indicators through hydrogel technology not only enhances food safety but also contributes to reducing food waste by enabling more informed decision-making regarding product consumption.

**2. Contaminant Detection**: Hydrogels tailored for contaminant detection represent a significant advancement in food safety technology, leveraging the unique properties of these materials to identify harmful substances with high specificity. By integrating antibodies or aptamers—molecules designed to bind selectively to specific targets—into the hydrogel matrix, these systems can effectively recognize pathogens, pesticides, and heavy metals present in food products. For instance, hydrogels functionalized with antibodies against pathogens like E. coli or Salmonella can undergo measurable changes in their optical or mechanical properties upon binding to the target contaminants. This responsiveness may manifest as alterations in fluorescence intensity, colorimetric changes, or shifts in mechanical stiffness, providing a rapid and visual indication of contamination. The advantages of using hydrogels in this context include their tunable swelling properties and the potential for real-time monitoring, which can be crucial in ensuring food safety during storage and transportation [159]. However, the development of effective hydrogel-based sensors poses challenges such as maintaining the stability and activity of the incorporated biomolecules over time and under various environmental conditions. Additionally, achieving high specificity while minimizing cross-reactivity with non-target substances is critical for ensuring accurate results. Advances in nanotechnology and materials science are being explored to enhance the performance of these hydrogels, including the incorporation of nanomaterials that could amplify the detection signal. Overall, the use of hydrogels for contaminant detection not only offers a promising solution for safeguarding public health but also has the potential to enhance consumer confidence in food products through transparent safety monitoring.

**3. Nutrient Monitoring**: Hydrogels can also monitor nutrient levels in food products. For example, hydrogels embedded with specific enzymes can detect the presence of sugars, amino acids, or vitamins by producing a measurable signal when these nutrients are present [160].

One notable advancement is the development of smart packaging incorporating hydrogel-based sensors. These packaging materials can monitor the condition of food in real time and provide consumers with visual indications of freshness or contamination. Such technology can significantly reduce food waste and enhance food safety [161]. Challenges in this field include ensuring the compatibility of hydrogel sensors with different types of food matrices and packaging materials. The stability and reliability of these sensors under various storage and transportation conditions are also critical factors that need to be addressed [162]. 

Future research should aim to develop biodegradable and edible hydrogel sensors that can be safely ingested with food, providing an added layer of safety and convenience. Enhancing the sensitivity and specificity of these sensors for a wider range of contaminants and quality indicators will further improve their utility. Moreover, integrating these sensors with digital tracking and traceability systems can enhance food supply chain transparency and safety [163]. 

### 4.5. Overall Challenges and Future Perspectives

Hydrogel-based sensors hold great promise across various fields, from medical diagnostics to environmental monitoring and food quality control. However, several challenges must be addressed to fully realize their potential.

**Material Challenges**

One of the primary challenges is the stability and durability of hydrogels under different conditions. Hydrogels can degrade or lose functionality over time, especially when exposed to harsh environmental conditions or biological fluids. Developing hydrogels with enhanced mechanical properties, self-healing capabilities, and long-term stability is essential [164]. 

**Sensitivity and Specificity**

Ensuring the sensitivity and specificity of hydrogel-based sensors is another significant challenge. Non-specific binding and interference from other substances can affect the accuracy of the sensors. Advances in materials science, such as the development of nanocomposite hydrogels and the incorporation of selective binding elements like aptamers and antibodies, are crucial to addressing these issues [165]. 

**Integration with Technology**

Integrating hydrogel sensors with advanced technologies like microfluidics, wireless communication, and artificial intelligence can enhance their functionality and ease of use. These integrations can enable real-time monitoring, data analysis, and remote diagnostics. However, achieving seamless integration while maintaining sensor performance and reliability is a complex task [166].

**Cost and Scalability**

The cost and scalability of hydrogel-based sensors are important considerations for widespread adoption. Developing cost-effective manufacturing processes and ensuring the scalability of sensor production without compromising quality is essential for their commercial viability [167]. 

**Regulatory and Ethical Considerations**

The regulatory landscape for hydrogel-based sensors, especially in medical and food applications, is evolving. Ensuring compliance with regulatory standards and addressing ethical concerns related to data privacy and security are critical for gaining public trust and acceptance [168]. 

**Future Perspectives**

The future of hydrogel-based sensors is bright, with numerous possibilities for innovation and application. Research should focus on developing multifunctional hydrogels that can simultaneously detect multiple analytes and provide therapeutic benefits. Enhancing the biocompatibility and environmental sustainability of hydrogels through the use of natural and biodegradable materials will be crucial [169,170]. 

Additionally, integrating hydrogel sensors with wearable and implantable devices can revolutionize healthcare by enabling continuous health monitoring and personalized treatment. In environmental and industrial applications, smart hydrogel sensors can contribute to more effective pollution control and food safety management [14,171]. So, interdisciplinary collaboration between material scientists, engineers, biologists, and healthcare professionals will be essential to overcoming current challenges and unlocking the full potential of hydrogel-based sensors.

## 5. Cross-Disciplinary Innovations

Hydrogels have emerged as a pivotal material in various fields due to their unique properties such as high water content, biocompatibility, and tunable mechanical properties. This section explores the integration of hydrogels in wearable devices, hydrogel composites and hybrid systems, the synergy between tissue engineering and biosensing, and their applications in personalized medicine.

### 5.1. Integration of Hydrogels in Wearable Devices

**Figure 12:**
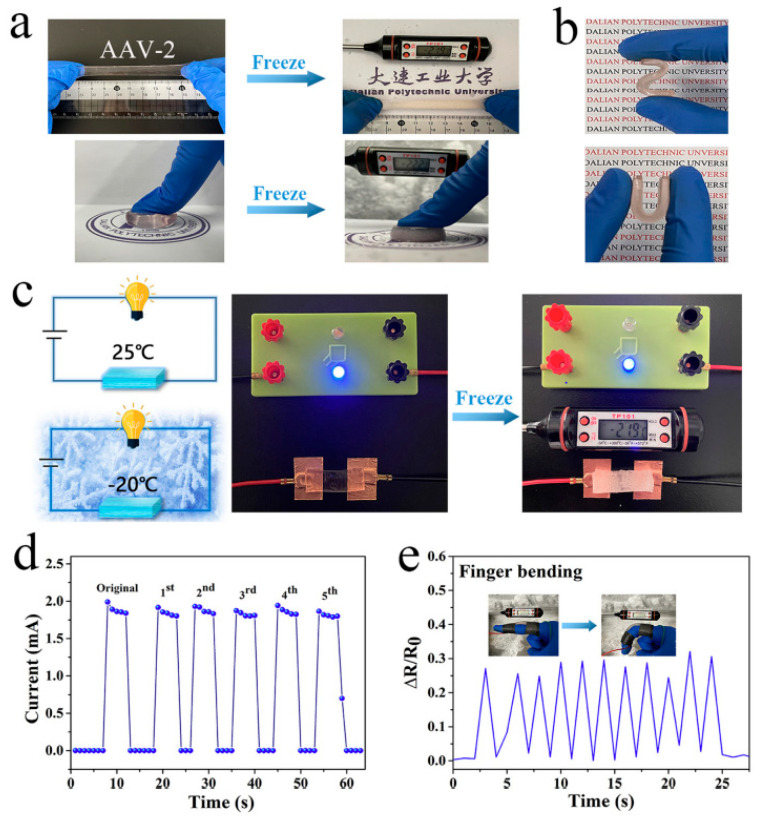
(**a**) Tensile and compressive properties of AAV-2 hydrogel before and after freezing, (**b**) photographs showing AAV-2 hydrogel bent into ‘2’ and ‘U’ shapes post-freezing, (**c**) electrical circuit using AAV-2 hydrogel as a conductor at 25 °C and −20 °C, (**d**) current response during the healing cycle of a cut in frozen AAV-2 hydrogel (repeated 5 times), (**e**) real-time finger bending response monitored by AAV hydrogel at −20 °C. Reprinted with permission from [172].

Wearable devices are transforming healthcare by providing continuous monitoring and real-time data collection. The integration of hydrogels into these devices offers several advantages, including flexibility, biocompatibility, and enhanced sensitivity for various bio-signals. Figure 12 provides a comprehensive analysis of the AAV-2 hydrogel’s performance under various conditions. Panel (a) demonstrates the tensile and compressive strength of the hydrogel before and after freezing, indicating its mechanical robustness. Panel (b) features images of the hydrogel bent into ‘2’ and ‘U’ shapes after freezing, showcasing its flexibility and shape retention. Panel (c) illustrates the use of AAV-2 hydrogel as a conductor in an electrical circuit, with performance measured at 25 °C and −20 °C. Panel (d) details the current response during the cut-healing cycle in frozen AAV-2 hydrogel, highlighting its electrical recovery capability. Finally, panel (e) captures the real-time response of finger bending monitored by the hydrogel at −20 °C, emphasizing its suitability for dynamic and low-temperature applications [172]. 

**Hydrogels in Biosensors**

Hydrogels are used in wearable biosensors for their ability to interact with biological fluids and transduce biochemical reactions into readable signals. For instance, glucose sensors incorporating hydrogels embedded with glucose oxidase can detect blood sugar levels by measuring the electrical signal generated from the enzyme-substrate reaction [173]. These sensors are crucial for diabetic patients who require continuous glucose monitoring.

**Skin-Compatible Electronics**

The soft and flexible nature of hydrogels makes them ideal for creating skin-compatible electronics. These hydrogels can be used to develop electrodes that adhere comfortably to the skin and can stretch and flex with body movements without causing discomfort or irritation. This feature is particularly useful for long-term health monitoring and for devices that need to conform to irregular skin surfaces [174].

**Sweat and Tear Sensors**

Sweat and tear are accessible bodily fluids that can provide valuable information about a person’s health status. Hydrogel-based sensors can be designed to detect various biomarkers in these fluids, such as electrolytes, metabolites, and proteins. For example, hydrogels that change color or fluorescence in response to pH changes or the presence of specific ions can be used to monitor hydration levels or electrolyte balance [175]. 

While hydrogel-based wearable devices offer many benefits, there are challenges to address. Ensuring the durability and longevity of these devices under daily wear conditions is crucial. Additionally, developing hydrogels that can maintain their functionality in the presence of sweat and other body fluids over extended periods is necessary [176]. Future research should focus on creating multifunctional hydrogels that can simultaneously monitor multiple health parameters. Integrating these sensors with wireless technology and mobile applications will enable more comprehensive health monitoring and personalized healthcare solutions [177]. 

### 5.2. Hydrogel Composites and Hybrid Systems

Hydrogel composites and hybrid systems represent a significant advancement in material science, offering enhanced mechanical properties, functionality, and applicability in various fields, including medicine, environmental science, and engineering.

**Composite Hydrogels**

Composite hydrogels are formed by incorporating different materials such as nanoparticles, fibers, or polymers into the hydrogel matrix. These composites exhibit improved mechanical strength, thermal stability, and responsiveness. For instance, incorporating carbon nanotubes or graphene into hydrogels can enhance their electrical conductivity, making them suitable for use in bioelectronics and sensors [178]. 

**Hybrid Hydrogels**

Hybrid hydrogels combine the properties of two or more types of hydrogels to achieve specific functionalities. These hybrids can respond to multiple stimuli, such as pH, temperature, and light, making them versatile for various applications. For example, hybrid hydrogels that respond to both pH and temperature changes can be used in drug delivery systems to release medication in a controlled manner based on the local environment [179]. 

**Applications in Environmental Monitoring**

Hydrogel composites and hybrids are being explored for environmental monitoring. For instance, hydrogels with embedded magnetic nanoparticles can be used to detect and remove heavy metal ions from water. These hydrogels can be easily manipulated using an external magnetic field, allowing for efficient pollutant removal and sensor regeneration [180]. The development of hydrogel composites and hybrids faces challenges such as ensuring uniform distribution of the incorporated materials and maintaining the biocompatibility of the resulting composite. Additionally, scaling up the production of these advanced materials while maintaining their unique properties is a significant hurdle [181]. 

Future research should focus on developing eco-friendly and sustainable hydrogel composites. Exploring the use of natural materials and biopolymers in creating these composites will enhance their environmental compatibility. Additionally, integrating these materials with advanced manufacturing techniques such as 3D printing can lead to the development of complex structures with tailored properties for specific applications [182]. 

### 5.3. Synergy between Tissue Engineering and Biosensing

The intersection of tissue engineering and biosensing represents a groundbreaking area of research with the potential to revolutionize healthcare by providing advanced diagnostic and therapeutic solutions.

**Scaffold-based Biosensors**

In tissue engineering, hydrogels are commonly used as scaffolds to support cell growth and tissue development. By integrating biosensing elements into these scaffolds, researchers can create systems that monitor the physiological state of the engineered tissues in real time. For example, sensors that detect changes in pH, oxygen levels, or glucose concentration can provide valuable information about the health and functionality of the tissue constructs [183]. 

**Real-time Monitoring of Tissue Growth**

Hydrogel-based biosensors can be used to monitor tissue growth and development in real time. This capability is crucial for ensuring the success of tissue engineering applications, such as organ transplantation or wound healing. Sensors embedded in the hydrogel scaffolds can detect biochemical and biophysical changes, providing insights into the tissue maturation process and enabling timely interventions if necessary [184]. 

**Drug Delivery and Monitoring Systems**

Hydrogels can be engineered to serve dual functions as both drug delivery vehicles and biosensors. These systems can release therapeutic agents in response to specific physiological triggers while simultaneously monitoring the treatment’s efficacy. For instance, a hydrogel that releases anti-inflammatory drugs in response to elevated levels of inflammatory markers can also provide feedback on the local inflammation status [185]. 

One of the main challenges in integrating biosensing capabilities into tissue engineering scaffolds is ensuring the stability and functionality of the sensors over extended periods. Additionally, the sensors must be biocompatible and not interfere with the tissue growth process [186]. 

Future research should aim to develop smart hydrogel scaffolds with integrated sensing and actuation capabilities. These scaffolds could respond to environmental changes by releasing drugs or growth factors to promote tissue healing and regeneration. Additionally, leveraging advances in nanotechnology and bioelectronics will enhance the sensitivity and functionality of these integrated systems [187]. 

### 5.4. Personalized Medicine Applications

Personalized medicine aims to tailor medical treatment to the individual characteristics of each patient. Hydrogel-based sensors and systems play a crucial role in achieving this goal by enabling precise and real-time monitoring of various biomarkers and physiological parameters.

**Customized Drug Delivery**

Hydrogel-based drug delivery systems can be designed to release medication at specific rates and times, tailored to the patient’s needs. These systems can respond to physiological signals such as pH, temperature, or specific biomarker levels, ensuring that the drug is released only when needed. For example, a hydrogel that releases insulin in response to elevated glucose levels can provide more precise control of blood sugar levels in diabetic patients [188].

**Point-of-Care Diagnostics**

Hydrogel-based sensors can be used in point-of-care diagnostic devices to provide rapid and accurate detection of various biomarkers. These devices can be used to monitor chronic diseases, detect infections, or assess the effectiveness of treatments. For instance, a hydrogel sensor that detects specific cancer biomarkers in body fluids can provide early diagnosis and enable timely treatment interventions [189]. 

**Personalized Health Monitoring**

Wearable hydrogel-based devices can continuously monitor various physiological parameters, providing valuable data for personalized health management. These devices can track metrics such as heart rate, hydration levels, and metabolic changes, enabling individuals to make informed decisions about their health and lifestyle [190]. 

Personalized medicine applications of hydrogel-based systems face challenges such as ensuring the specificity and sensitivity of the sensors, biocompatibility with diverse biological environments, maintaining stability and reproducibility of sensor performance over time, and integrating these systems seamlessly with existing medical technologies for accurate and reliable diagnostics and treatments. One of the primary challenges in personalized medicine applications is ensuring the specificity and sensitivity of hydrogel-based sensors. These sensors must accurately detect and quantify biomarkers or physiological parameters relevant to individual health conditions without cross-reactivity or interference from other substances. Advances in nanotechnology, such as the development of nanostructured hydrogels or nanocomposite materials, hold promise for enhancing sensor performance and selectivity [191]. 

Additionally, ensuring the biocompatibility of hydrogel-based devices and systems is crucial for their safe and effective use in clinical settings. The materials used in these devices must not elicit adverse immune responses or tissue reactions, especially in long-term applications. Research efforts are focusing on developing bio inert and biodegradable hydrogel formulations that minimize the risk of inflammatory responses and promote tissue integration [192]. 

**Integration of Data Analytics**

The integration of advanced data analytics, including artificial intelligence (AI) and machine learning algorithms, is essential for extracting actionable insights from the vast amount of data generated by hydrogel-based sensors. These analytics can help in predicting disease progression, optimizing treatment strategies, and enabling early intervention based on real-time health monitoring data [193]. 

**Personalized Therapeutics**

Hydrogel-based platforms enable the delivery of personalized therapeutics tailored to individual patient profiles. By incorporating stimuli-responsive elements into hydrogel matrices, drug release can be controlled in response to specific physiological cues. For example, temperature-sensitive hydrogels can release drugs in response to fever or inflammation, providing targeted therapy while minimizing systemic side effects [194]. 

**Regulatory Considerations**

The regulatory landscape for personalized medicine and medical devices is evolving, requiring adherence to stringent standards for safety, efficacy, and data privacy. Regulatory agencies worldwide are actively developing guidelines to ensure the ethical use and regulatory compliance of hydrogel-based technologies in healthcare applications [195]. 

**Future Perspectives**

The future of hydrogel-based technologies in cross-disciplinary applications is promising, driven by ongoing advancements in material science, biotechnology, and digital health innovations. Collaborative efforts among researchers, clinicians, engineers, and regulatory bodies will be crucial in overcoming current challenges and realizing the full potential of hydrogel-based systems.

## 6. Conclusions

Hydrogel-based sensors represent a significant leap in the field of material science, offering versatile applications across healthcare, environmental monitoring, and industry. This section provides a comprehensive summary of key findings, discusses the potential impact on various sectors, and outlines future research directions to advance hydrogel technology further.

### 6.1. Summary of Key Findings

Hydrogels have been extensively studied for their unique properties, such as high water content, flexibility, biocompatibility, and tunable mechanical properties. These characteristics make them suitable for a wide range of applications, particularly in the development of advanced sensors.

**Versatility in Sensing Mechanisms**

Hydrogel-based sensors employ various mechanisms to detect environmental and biological stimuli, including optical, electrochemical, and piezoelectric sensing. Optical sensors leverage the transparency and refractive properties of hydrogels to monitor changes in light absorption or emission. Electrochemical sensors benefit from the ionic conductivity of hydrogels to measure electrical signals related to biochemical reactions. Piezoelectric sensors exploit the mechanical flexibility of hydrogels to generate electrical signals in response to pressure or deformation.

**Integration with Wearable Devices**

The integration of hydrogels into wearable devices has demonstrated significant potential in health monitoring. Hydrogels’ softness and flexibility allow for comfortable skin contact, making them ideal for continuous monitoring of physiological parameters such as glucose levels, hydration, and vital signs. These wearable sensors provide real-time data, enhancing the management of chronic conditions and enabling prevention.

**Environmental and Industrial Applications**

In environmental monitoring, hydrogel-based sensors have been employed to detect pollutants and contaminants. Their ability to respond to chemical changes in water or air makes them suitable for monitoring heavy metals, organic compounds, and other environmental toxins. In the food industry, hydrogel sensors have been used for real-time monitoring of food quality and safety, detecting spoilage, and ensuring regulation.

**Advances in Tissue Engineering and Biosensing**

The synergy between tissue engineering and biosensing has led to innovative applications in regenerative medicine. Hydrogel scaffolds integrated with biosensors can monitor tissue growth, detect infection, and release therapeutic agents in a controlled manner. This integration enhances the functionality of engineered tissues and improves patient outcomes.

### 6.2. Potential Impact on Healthcare and Industry

Hydrogel-based sensors have transformative potential across multiple sectors, particularly healthcare and industry. Their unique properties and versatile applications could lead to significant advancements in diagnostics, personalized medicine, environmental protection, and industrial processes.

**Healthcare Advancements**

In healthcare, hydrogel-based sensors can revolutionize diagnostics and patient monitoring. Continuous glucose monitoring systems, for instance, can provide diabetic patients with real-time data, reducing the risk of complications and improving disease management. Additionally, hydrogel sensors that monitor hydration and electrolyte levels can help prevent dehydration and electrolyte imbalances, particularly in vulnerable populations such as the elderly and athletes.

Personalized medicine stands to benefit greatly from hydrogel technology. By tailoring drug delivery systems to an individual patient’s needs, hydrogel-based devices can optimize therapeutic outcomes and minimize side effects. For example, hydrogels that release drugs in response to specific biomarkers can provide targeted therapy for conditions like cancer, where localized treatment can significantly reduce systemic.

**Environmental Monitoring**

Hydrogel sensors offer effective solutions for environmental monitoring, particularly in detecting and mitigating pollution. Their sensitivity to chemical changes makes them ideal for monitoring water quality and detecting contaminants such as heavy metals and pesticides. These sensors can provide real-time data, enabling prompt responses to environmental hazards and contributing to better resource management and environmental protection.

**Industrial Applications**

In the industrial sector, hydrogel sensors can enhance quality control processes and improve safety standards. For example, in the food industry, hydrogel sensors can monitor the freshness and safety of products, ensuring compliance with health regulations and reducing food waste. Additionally, in manufacturing, hydrogel-based sensors can monitor the structural integrity of materials and detect the presence of hazardous substances, enhancing overall safety and efficiency.

### 6.3. Future Research Directions

The future of hydrogel-based sensors lies in addressing current challenges and exploring new frontiers in their application. Key areas for future research include enhancing sensor performance, expanding the range of detectable analytes, and developing sustainable and biocompatible materials.

**Enhancing Sensor Performance**

Improving the sensitivity, specificity, and stability of hydrogel-based sensors is crucial for their widespread adoption. Advances in nanotechnology and material science can lead to the development of nanocomposite hydrogels with superior properties. For instance, incorporating nanomaterials such as carbon nanotubes or graphene can enhance the electrical conductivity and mechanical strength of hydrogels, making them more effective for various sensing applications.

**Expanding the Range of Detectable Analytes**

Expanding the range of detectable analytes will enable hydrogel-based sensors to address a broader spectrum of applications. Future research should focus on developing hydrogels that can detect multiple biomarkers simultaneously, providing comprehensive health monitoring and environmental assessments. Multi-functional hydrogels that respond to different stimuli, such as pH, temperature, and chemical concentrations, can offer versatile solutions for complex diagnostic and monitoring needs.

**Developing Sustainable and Biocompatible Materials**

Sustainability and biocompatibility are critical considerations for the future development of hydrogel-based sensors. Research efforts should aim to create eco-friendly hydrogels using natural polymers and biodegradable materials. These sustainable hydrogels should not only meet performance requirements but also minimize environmental impact and ensure safe disposal. Additionally, developing biocompatible hydrogels that do not elicit adverse immune responses will be essential for medical applications, particularly in long-term implants and wearable devices.

**Integrating Advanced Data Analytics**

The integration of advanced data analytics, such as artificial intelligence (AI) and machine learning, with hydrogel-based sensors can unlock new possibilities in real-time data interpretation and predictive analysis. These technologies can help identify patterns, predict disease progression, and optimize treatment plans based on continuous monitoring data. Collaborative efforts between material scientists, data scientists, and healthcare professionals will be crucial in realizing the full potential of smart hydrogel-based systems.

**Addressing Regulatory and Ethical Challenges**

As hydrogel-based sensors move towards clinical and commercial use, addressing regulatory and ethical challenges will be paramount. Ensuring compliance with safety standards, protecting patient privacy, and establishing ethical guidelines for data usage are essential steps for the successful implementation of these technologies. Engaging with regulatory bodies and stakeholders will facilitate the development of frameworks that support innovation while safeguarding public health and safety.

The final conclusion of Hydrogel-based sensors has demonstrated remarkable potential across various fields, offering innovative solutions for health monitoring, environmental protection, and industrial applications. The ongoing advancements in material science, biotechnology, and data analytics will continue to drive the development of hydrogel-based technologies, making them more effective, versatile, and sustainable. By addressing current challenges and exploring new research directions, the scientific community can unlock the full potential of hydrogel-based sensors, leading to significant improvements in healthcare, environmental management, and industrial processes.

## Figures and Tables

**Table 1:** Overview of hydrogel synthesis processes, properties, and applications.

Hydrogel Type	Hydrogel	Synthesis Process	Outcome	Advantages	Disadvantages	Results	Ref.
**Natural**	**Alginate Hydrogel**	**Ionotropic Gelation**	Biocompatible, high water content	Easy to process, good for cell encapsulation	Limited mechanical strength, degradation by ions	Effective for drug delivery and tissue engineering	[27,28]
**Natural**	**Collagen Hydrogel**	**Enzymatic Cross-Linking**	Supports cell adhesion and proliferation	Natural ECM mimic, excellent biocompatibility	High cost, variability in source	Suitable for wound healing and tissue engineering	[31]
**Natural**	**Chitosan Hydrogel**	**Chemical Cross-Linking**	Biodegradable, antimicrobial properties	Natural origin, good for drug delivery	Limited mechanical strength, low gel stability	Useful for wound dressing and controlled release	[28]
**Natural**	**Hyaluronic Acid Hydrogel**	**Photocross-Linking**	High biocompatibility, supports cell migration	Hydrophilic, good for cartilage regeneration	Degradation over time, expensive	Effective for cartilage repair and regenerative medicine	[27]
**Synthetic**	**Polyethylene Glycol (PEG) Hydrogel**	**Photopolymerization**	Tunable mechanical and chemical properties	Precise control over structure, low toxicity	Requires UV light, potential cytotoxicity	Effective for 3D bioprinting and controlled drug release	[29,30]
**Synthetic**	**Poly(N-isopropylacrylamide) (PNIPAAm) Hydrogel**	**Free-Radical Polymerization**	Temperature-sensitive, high swelling ratio	Stimulus-responsive, excellent for biosensing	Limited biocompatibility, complex synthesis	Useful for smart drug delivery systems and biosensing	[29]
**Synthetic**	**Poly(vinyl alcohol) (PVA) Hydrogel**	**Freeze–Thaw Cycling**	High flexibility, good water retention	Mechanical properties vary with cycles	Limited biological compatibility	Effective for tissue engineering and as a matrix for cell cultures	[28]
**Synthetic**	**Polyacrylamide (PAAm) Hydrogel**	**Chemical Cross-Linking**	High mechanical strength, easy to fabricate	Not biodegradable, potential toxicity	Used for gel electrophoresis and tissue scaffolding		[31]
